# Maternal nutrition and its intergenerational links to non-communicable disease metabolic risk factors: a systematic review and narrative synthesis

**DOI:** 10.1186/s41043-021-00241-2

**Published:** 2021-04-26

**Authors:** Elizabeth Wilkins, Kremlin Wickramasinghe, Jessie Pullar, Alessandro R. Demaio, Nia Roberts, Karla-Maria Perez-Blanco, Katharine Noonan, Nick Townsend

**Affiliations:** 1 Centre on Population Approaches for NCD Prevention, University of Oxford, Oxford, UK; 2 VicHealth, Carlton, Victoria Australia; 3 Health Library, Nuffield Department of Population Health, University of Oxford, Oxford, UK; 4 Centre on Migration, Policy and Society (COMPAS), University of Oxford, Oxford, UK; 5 Department of Health, Western Australia, Perth, Australia; 6 Department for Health, University of Bath, Bath, BA2 7AY UK

## Abstract

**Background:**

Non-communicable diseases (NCDs) are the leading cause of death and disability globally, while malnutrition presents a major global burden. An increasing body of evidence suggests that poor maternal nutrition is related to the development of NCDs and their risk factors in adult offspring. However, there has been no systematic evaluation of this evidence.

**Methods:**

We searched eight electronic databases and reference lists for primary research published between 1 January 1996 and 31 May 2016 for studies presenting data on various dimensions of maternal nutritional status (including maternal exposure to famine, maternal gestational weight gain (GWG), maternal weight and/or body mass index (BMI), and maternal dietary intake) during pregnancy or lactation, and measures of at least one of three NCD metabolic risk factors (blood pressure, blood lipids and blood glucose) in the study population of offspring aged 18 years or over. Owing to high heterogeneity across exposures and outcomes, we employed a narrative approach for data synthesis (PROSPERO= CRD42016039244, CRD42016039247).

**Results:**

Twenty-seven studies from 10 countries with 62,607 participants in total met our inclusion criteria. The review revealed considerable heterogeneity in findings across studies. There was evidence of a link between maternal exposure to famine during pregnancy with adverse blood pressure, blood lipid, and glucose metabolism outcomes in adult offspring in some contexts, with some tentative support for an influence of adult offspring adiposity in this relationship. However, the evidence base for maternal BMI, GWG, and dietary intake of specific nutrients during pregnancy was more limited and revealed no consistent support for a link between these exposures and adult offspring NCD metabolic risk factors.

**Conclusion:**

The links identified between maternal exposure to famine and offspring NCD risk factors in some contexts, and the tentative support for the role of adult offspring adiposity in influencing this relationship, suggest the need for increased collaboration between maternal nutrition and NCD sectors. However, in view of the current scant evidence base for other aspects of maternal nutrition, and the overall heterogeneity of findings, ongoing monitoring and evaluation using large prospective studies and linked data sets is a major priority.

**Supplementary Information:**

The online version contains supplementary material available at 10.1186/s41043-021-00241-2.

## What is already known and what this review adds

We identified several non-systematic, narrative reviews that focused largely on the developmental origins of metabolic risk factors and NCD outcomes [1–6]. In terms of reviews that discussed evidence, two supported a link between maternal undernutrition and overnutrition and offspring NCDs. In contrast, a recent systematic review of maternal overnutrition found no consistent support for a link with cardiovascular risk factors in adult offspring [7].

To our knowledge, this is the first systematic review to examine the relationship between various dimensions of maternal nutritional status during pregnancy or lactation and blood pressure, blood lipids, and glucose metabolism in adult offspring. Our findings support an association between maternal gestational exposure to famine and offspring metabolic NCD risk factors in some contexts, but reveal no consistent support for a relationship between maternal gestational weight gain (GWG), maternal weight or body mass index (BMI), or maternal dietary intake and offspring NCD risk factors. Our findings also show some tentative support for an influence of adult offspring adiposity in the maternal exposure to famine—offspring NCD risk factor relationship. Importantly, our review exposes the paucity of the current evidence base and considerable heterogeneity across studies.

## Background

Malnutrition represents one of the greatest global health challenges of our time. In 2015, approximately 462 million adults worldwide were underweight and 264 million women of reproductive age were affected by iron-amenable anaemia, while 1.9 billion adults were either overweight or obese [8]. This co-existence of undernutrition and over-nutrition, or ‘double burden of malnutrition’, is of particular concern in lower middle-income countries (LMIC).

Meanwhile, unprecedented rates of economic and income growth, urbanisation, and globalisation have led to a rapid rise in the burden of non-communicable diseases (NCDs), principally cardiovascular disease (CVD), cancers, chronic respiratory diseases, and diabetes. NCDs are now the world’s leading cause of death and disability, responsible for 71% of deaths and 60% of disability-adjusted life years (DALYs) globally in 2015, with the burden of premature deaths from these diseases also felt disproportionately in low- and lower middle-income countries (LLMIC) [9].

Unhealthy diets constitute the largest behavioural risk factor for NCDs, with 30% of global NCD deaths and 18% of NCD DALYs attributable to dietary risk factors [9]. Mounting clinical, experimental, and epidemiological evidence suggests that the influence of dietary nutrition on the development of NCDs operates at multiple stages throughout the life-course, and moreover, between generations. Specifically, it seems that sub-optimal maternal nutrition before and during pregnancy can induce changes in foetal development that predispose offspring in later life to immediate NCD metabolic risk factors such as raised blood pressure, high blood lipid levels, impaired glucose tolerance, and overweight/obesity, and in turn NCDs [1, 2, 5, 6, 10].

A number of narrative reviews have explored this relationship, with the focus on the underlying theory and potential mechanisms [1–6]. Discursive reviews of the evidence on maternal *undernutrition* during pregnancy have suggested a link with offspring risk of NCDs [5, 6]. Reviews of the evidence on maternal *overnutrition* have been more mixed, with some finding a link with the development of offspring NCDs and risk factors [5, 6], and others finding no consistent associations [7]. To our knowledge, no study has systematically reviewed the relationship between various dimensions of maternal nutritional status during pregnancy and lactation and the development of metabolic risk factors for NCDs in adult offspring.

Such a review is needed to identify critical windows and intervention focus points for NCD prevention, and to inform the future work of maternal nutrition and NCD sectors. It is also an appropriate time to conduct such a review, given the declaration by the United Nations General Assembly of 2016 to 2025 as the *Decade of Action on Nutrition* [11], the respective SDG targets to end all forms of malnutrition and reduce premature NCD mortality by one third by 2030 [12], and the current international focus on collaboration across different sectors. Accordingly, this paper aims to systematically assess the evidence on the relationship between maternal nutritional status during pregnancy and lactation and three metabolic NCD risk factors—elevated blood pressure, high blood lipid levels, and impaired glucose tolerance—in adult offspring.

## Methods

### Search strategy and selection criteria

We conducted a systematic review following PRISMA guidelines (Additional file 1) [13]: PROSPERO registration numbers: CRD42016039244, CRD42016039247. We searched CINAHL, Cochrane Database of Systematic Reviews, Cochrane Register of Controlled Trials, Database of Abstracts of Reviews of Effects, MEDLINE, EMBASE, Web of Science, and Global Health for all studies that included primary data published between 1 January 1996 and 31 May 2016. We also reviewed the reference lists of reviews identified during screening and of included papers. We did not review grey literature. We used English search terms (Additional file 2) but placed no restrictions on the publication language, populations assessed, or study design.

The study population was adult offspring (age 18 years and over). The exposure variable was maternal nutritional status during pregnancy or lactation. We defined maternal nutritional status broadly to include maternal exposure to famine (measured by proxy indicators, such as the official daily rations for the population aged 21 years and over combined with birth date); maternal gestational weight gain (GWG); maternal weight and/or body mass index (BMI); and maternal dietary intake. The outcome variable was NCD metabolic risk factors in adult offspring. Specifically, we studied three NCD metabolic risk factors: blood pressure, blood lipid levels/metabolism, and blood glucose levels/metabolism (the specific outcome indicators for each study are presented in Table 1). We did not study outcome measures of offspring overweight/obesity, taking the view that the very large volume of studies on this outcome merited a separate, future systematic review.

**Table 1:** Summary of study characteristics

	Site	Study design	Number of participants	Age of adult offspring at follow-up	Exposure	Outcome
De Rooij et al. [14]	Netherlands	Cohort	672	58	Famine	Glucose metabolism (FPG, 120 minute glucose)
De Rooij et al. [15]	Netherlands	Cohort	783	58	Famine	Blood pressure (SBP, DBP) Blood lipids Glucose metabolism
Huang et al. [16]	China	Cohort	33247 (total *N*=35025)	32	Famine	Blood pressure (prevalence of hypertension)
Li et al. [17]	China		2959 (total *N*=7874)	43–45	Famine	Glucose metabolism: FPG, prevalence of hyperglycemia
Ravelli et al. [18]	Netherlands	Cohort	702	51–55	Famine	Glucose metabolism (FPG, 30 minute glucose, 120 minute glucose) Blood lipids
Roseboom et al. [19]	Netherlands	Cohort	739	51–55	Famine	Blood pressure (SBP, DBP)
Roseboom et al. [20]	Netherlands	Cohort	704	50	Famine	Blood lipids (total cholesterol, LDL, HDL, LDL:HDL, Apo-A1, Apo-B)
Stanner et al. [21]	Russia	Cross-sectional	549	52–53	Famine	Blood pressure: SBP, DBP
Stein et al. [22]	Netherlands	Cohort	971	56–62	Famine	Blood pressure (SBP, DBP, prevalence of hypertension)
Wang et al. [23]	China	Cross-sectional	2420 (total *N*=6445)	52–53	Famine	Blood pressure: (SBP, DBP, prevalence of hypertension) Blood lipids Glucose metabolism
Zheng et al. [24]	China	Cross-sectional	3696 (total *N*=5040)	44–51	Famine	Blood pressure (SBP, DBP, prevalence of hypertension) Blood lipids Glucose metabolism
Loos et al. [25]	Belgium	Cross-sectional	800	18–34	GWG, ppBMI	Glucose metabolism (FPG, fasting plasma proinsulin, fasting plasma insulin, HOMA-IR, HOMA)
Mamun et al. [26]	Australia	Cohort	2271	21	GWG	Blood pressure (SBP, DBP)
Hochner et al. [27]	Israel	Cohort	1130	32	GWG, ppBMI	Blood pressure (SBP, DBP) Blood lipids Glucose metabolism
Hrolfsdottir et al. [28]	Denmark	Cohort	308	19–20	GWG	Blood pressure (SBP, DBP) Blood lipids Glucose metabolism
Mi et al. [29]	China	Cross-sectional	627	41–47	GWG BMI	Blood pressure(SBP, DBP, prevalence of hypertension) Blood lipids Glucose metabolism
Scheers-Andersson et al. [30]	Sweden	Cohort	9816	18.3	GWG	Blood pressure (SBP, DBP, prevalence of hypertension)
Webb et al. [31]	Guatemala	Longitudinal	450	21–29	GWG, BMI, dietary intake (protein and micronutrient supplementation)	Blood pressure (SBP, DBP)
Hochner et al. [27]	Israel	Cohort	1130	32	GWG, ppBMI	Blood pressure (SBP, DBP) Blood lipids Glucose metabolism
Loos et al. [25]	Belgium	Cross-sectional	800	18–34	GWG, ppBMI	Glucose metabolism (FPG, fasting plasma proinsulin, fasting plasma insulin, HOMA-IR, HOMA)
Mi et al. [29]	China	Cross-sectional	627	41–47	GWG, BMI	Blood pressure (SBP, DBP) Blood lipids Glucose metabolism
Webb et al. [31]	Guatemala	Longitudinal	450	21–29	GWG, BMI, dietary intake (protein and micronutrient supplementation)	Blood pressure (SBP, DBP)
Campbell et al. [32]	UK	Cohort	253	40.6	Maternal dietary intake: protein, animal protein, fat, carbohydrate, calcium, vitamin A, thiamine, riboflavin, niacin, vitamin C	Blood pressure (SBP, DBP)
Conlisk et al. [33]	Guatemala	Longitudinal	429	24.4	Maternal dietary intake: protein and micronutrient supplementation	Glucose metabolism (FPG)
Danielsen et al. [34]	Denmark	Cohort	428	20	Maternal dietary intake: GI, GL	Blood pressure (SBP, DBP) Blood lipids Glucose metabolism
Macleod et al. [35]	UK	RCT	118	22–23	Maternal dietary intake: protein and carbohydrate supplementation	Blood pressure (SBP, DBP) Blood lipids Glucose metabolism
Roseboom et al. [36]	Netherlands	Cohort	739	50	Maternal dietary intake: protein/carbohydrate ratio	Blood pressure (SBP, DBP)
Rytter et al. [37]	Denmark	RCT	243	18–19	Maternal dietary intake: fish oil supplementation	Blood lipids (total cholesterol, LDL, HDL, TAG, Apo-A1, Apo-B)
Rytter et al. [38]	Denmark	RCT	180	19	Maternal dietary intake: fish oil supplementation	Blood pressure (SBP, DBP)
Rytter et al. [39]	Denmark	Cohort	443	19–20	Maternal dietary intake: fish oil supplementation	Blood pressure (SBP, DBP)
Shiell et al. [40]	UK	Cohort	626	27–30	Maternal dietary intake: meat and fish consumption plus low carbohydrate	Blood pressure (SBP, DBP)
Webb et al. [31]	Guatemala	Longitudinal	450	21–29	GWG, BMI, dietary intake (protein and micronutrient supplementation)	Blood pressure (SBP, DBP)

*GWG*, gestational weight gain; *BMI*, body mass index; *ppBMI*, pre-pregnancy body mass index; *GI*, GlycFPG = Fasting Plasma Glucose; *SBP*, systolic blood pressure; *DBP*, diastolic blood pressure; *HDL*, high-density lipoprotein; *LDL*, low-density lipoprotein; *TAG*, triacylglycerides; *HOMA-IR*, homeostatic model assessment for insulin resistance

We included records if they presented summary estimate data on one or more indicators of maternal nutritional status during pregnancy or lactation, and measures of at least one of the three NCD metabolic risk factors for offspring aged 18 years or over. We excluded studies if they did not include a measure of maternal nutritional status, or did not include a measure of one of the three NCD metabolic risk factors in adult offspring, were not primary research, or were conducted in animals. In addition, we did not include studies that focused on mothers with pre-existing medical conditions, or a diagnosis of preeclampsia or gestational diabetes during pregnancy, since we were interested in the link between mothers’ nutritional status and offspring NCD metabolic risk factors in the general population. A specific focus on the interaction between nutrition and underlying medical conditions in this relationship would have added a level of complexity due to the range of possible medical conditions.

Jessie Pullar (JP), Karla-Maria Perez-Blanco (KP), and Katharine Noonan (KN) independently screened the titles and abstracts of the initial 21,659 records. The Cohen’s *κ* statistic was calculated at 10% intervals (approximately every 2000 papers) to check percentage agreement. Once Cohen’s *κ* exceeded 0.75 (excellent agreement [41]), KP, and KN screened all remaining records. Uncertainties were brought to JP, Kremlin Wickramasinghe (KW), and Nick Townsend (NT), and disagreements were resolved by group consensus. Elizabeth Wilkins (EW) and JP then reviewed all records selected for full text review, resolving any uncertainties by group consensus with KW and NT.

### Data extraction

From each included full-text study, EW and JP extracted information on study type; nature of sample, including sample size and percentage male; offspring age; maternal nutrition exposure measures; offspring metabolic risk factor outcomes; and results. Ambiguities were resolved by group consensus. Where data were unclear or incomplete, the authors were contacted by email, and the study excluded if the information was unavailable.

We used a modified, design-specific versions of the Newcastle-Ottawa scale to assess the quality of included studies (Additional file 3). The scoring system was based on the selection of study groups, comparability of groups, ascertainment of exposure and outcome measures, and methods to control for confounders.

The main outcomes extracted were differences in blood pressure, blood lipid, and glucose metabolism indicators for offspring of mothers with different levels of a given nutritional indicator (see Table 1 for the specific outcome indicators for each study). We also planned, where possible, to investigate how the findings were influenced by the specific risk factor outcome, offspring sex, gestational timing of exposure, and adult offspring adiposity. We assessed within-study variability in our quality scoring when considering the repeatability of measuring instruments. Owing to the heterogeneity of the design and outcome measures of included studies, a meta-analysis was not conducted. Instead, narrative synthesis of data was conducted by EW, with studies grouped by outcome measure. We used Microsoft Excel to calculate simple descriptive statistics.

### Ethical clearance

Ethical clearance was not required as this is a systematic review of literature, and anonymised data were used throughout.

## Results

**Fig. 1:**
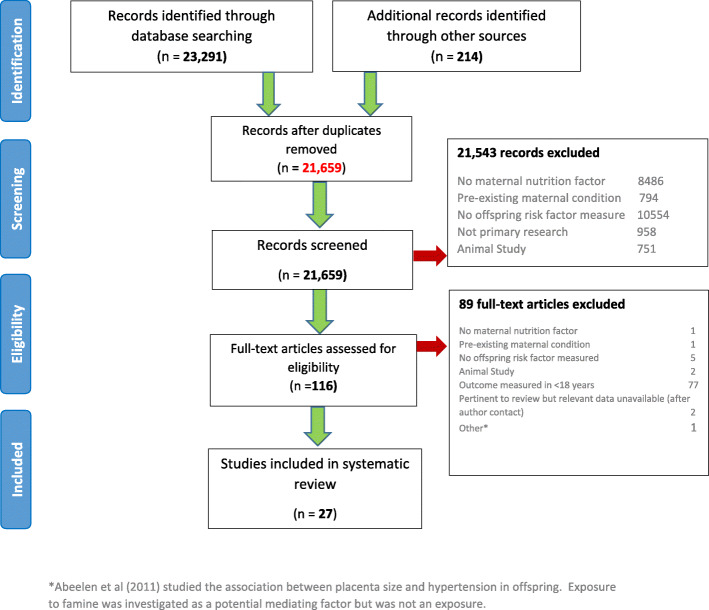
PRISMA flow diagram of systematic review results

We identified 23,291 records from our database search and 214 records from other sources, yielding 21,659 records after the removal of duplicates. Following initial screening of these 21,659 records, we reviewed 116 full-text articles, 27 of which met our inclusion criteria (Fig. 1).

The included studies were published between 1996 and 2015 and included 62,607 participants in total. Overall, ten countries were represented: seven from the World Health Organization’s (WHO) European Region, two from the Western Pacific Region, and one from the Region of the Americas. When classified by World Bank income group, there were seven high-income economies, two upper-middle-income economies, and one lower-middle-income economy. Seventeen studies were cohort studies, seven were case-control studies, and three were randomised-controlled trials (RCTs). In total, there were 16 high-quality and 11 medium-quality studies (Additional file 4), indicating a low risk of bias overall. Each of the included studies addressed one or more of four maternal nutrition exposures: maternal exposure to famine, maternal GWG, maternal weight or BMI, and maternal dietary intake. No studies measured maternal nutritional status during lactation. The characteristics of included studies are summarised in Table 1; more in-depth findings are presented in the Supplementary Table 1 (Additional file 5).

### Maternal exposure to famine

Eleven studies examined the association between maternal exposure to famine (measured by proxy indicators, such as the official daily rations for the population aged 21 years and over combined with birth date) and NCD metabolic risk factors in adult offspring. Six of these studies were of high quality and five were of medium quality. There were six cohort studies of the Dutch famine of 1944–1945, four studies of the Great Chinese famine of 1958–1961, and one study of the 1941–1944 Leningrad siege. Seven of the eleven studies reported on offspring blood pressure outcomes, five on blood lipid outcomes, and seven on glucose metabolism outcomes. Four of the eleven studies, including the single study of the Leningrad siege, found no significant differences in blood pressure [15, 16, 19, 21], blood lipids [21], or indicators of glucose metabolism [15, 21] between offspring exposed to famine in utero and unexposed offspring. Conversely, seven studies, five of the Dutch famine and two of the Chinese famine, revealed significantly higher blood pressure and/or prevalence of hypertension [22–24], adversely altered blood lipids [15, 20, 23, 24], and impaired glucose metabolism [14, 18, 23, 24] in fetally exposed versus non-exposed offspring. There was no clear evidence of a difference in the nature of the relationship for different NCD risk factor outcomes.

From the included studies, there was no clear evidence of differences by sex of the offspring. Of the four papers that stratified findings by sex, two studies—both of the Chinese famine—observed significantly higher blood pressure and blood lipids in exposed versus non-exposed female but not male offspring [23, 24]; one study of the Dutch famine found the reverse, namely significantly lower high-density lipoproteins (HDL), which is associated with higher NCD risk, in exposed versus not exposed male but not female offspring [15]; while another study of blood pressure outcomes in offspring of mothers exposed to the Dutch famine revealed no significant sex differences [22]. There was no clear evidence from the included studies of differences by timing of gestational exposure. Of the five studies—all of the Dutch famine—that stratified results by gestational timing of exposure, two found that offspring blood pressure outcomes were independent of the timing of gestational exposure to famine [14, 19], two found a more atherogenic blood lipid profile in offspring exposed to famine in early compared to later gestation [14, 20], and one found that offspring exposed to famine in later gestation had the highest rates of impaired glucose tolerance [18].

However, there was some tentative support for a role of adult offspring adiposity in influencing the maternal exposure to famine—offspring NCD risk factor relationship. Of the studies that measured the influence of adult offspring adiposity or indicators of postnatal nutritional abundance [15, 17, 18, 20–22], several supported a role of this variable in the relationship between maternal exposure to famine and NCD risk factors in adult offspring. A study of the Chinese famine found evidence that the association between foetal exposure to severe famine and the risk of adult hyperglycemia seemed to be exacerbated in subjects who consumed an energy-dense diet as adults [17]. A study of the Dutch famine found that the relationship between gestational exposure to famine and adult hypertension was attenuated following adjustment for adult waist circumference, with authors suggesting that this demonstrated a potential mediating role for this variable in the association [22], and another study of the Dutch famine found that while in utero exposure to famine was associated with glucose tolerance at all adult body mass indices, the highest plasma glucose concentrations were found in those exposed participants who became obese in adulthood [18]. It is also worth noting that while the study of the Leningrad Siege found no difference between subjects exposed to famine in utero versus infancy in glucose tolerance, insulin concentration, blood pressure, lipid concentration, or coagulation factors, the authors did find evidence, in female subjects, of a significantly stronger association between obesity and systolic and diastolic blood pressure in those subjects who had been exposed to famine in utero, which they interpreted as suggesting that foetal exposure to famine and adult obesity may operate in synergy to increase the risk of hypertension [21].

### Maternal gestational weight gain (GWG)

Seven studies examined the relationship between maternal GWG and NCD metabolic risk factors in adult offspring [25–31]. Four of these also examined the impact of maternal BMI [25, 27, 29, 31]. Six studies were of high quality and one was of medium quality. Six measured offspring blood pressure outcomes, three blood lipid outcomes, and four glucose metabolism outcomes. Overall, only two of the seven studies revealed a significant association between measures of maternal weight gain during pregnancy and offspring NCD metabolic risk factors [27, 28]. One of these—a Danish cohort study of 308 19–20-year-old males—found significant positive associations between GWG during the first 30 weeks of gestation (GWG30) and adult offspring systolic blood pressure (SBP), plasma insulin and homeostatic model assessment for insulin resistance (HOMA-IR) (for men only), and significant negative associations between GWG30 and adult offspring total cholesterol and *low-density lipoprotein* (LDL) cholesterol levels [28]. The other study found positive relationships between unadjusted maternal GWG and adult offspring blood pressure and triglyceride concentrations, although these associations were attenuated to non-significance following adjustment for adult offspring BMI [27]. The remaining five studies found no significant relationship between maternal GWG and offspring blood pressure [26, 29–31], blood lipids [29], or glucose metabolism [25, 29].

There was no clear difference in the results of included studies according to specific NCD metabolic risk factor outcome. Only two studies reported results stratified by sex, but the findings were not consistent. There was insufficient evidence from the included studies to draw conclusions about the role of gestational timing of exposure in the GWG-NCD risk factor relationship. Three studies explored the influence of offspring adiposity [26–28]. Of these, two cohort studies from Israel and Denmark respectively found that the significant positive associations between GWG and offspring NCD risk factors were significantly attenuated following adjustment for offspring adult BMI and leptin levels respectively [27, 28]. In the remaining Australian cohort study, the association between GWG and offspring blood pressure, while not significant in itself, was consistent in size with the association of maternal GWG with offspring BMI and of offspring BMI with their blood pressure, further supporting an influence of adult adiposity in the relationship [26].

### Maternal weight/body mass index (BMI)

Four studies investigated the association between maternal weight or BMI prior to or during pregnancy and offspring NCD risk factors [25, 27, 29, 31]. These studies examined maternal BMI as a continuous variable and did not analyse results for ‘low’ and ‘high’ maternal BMI specifically. All were of high quality. Three examined blood pressure outcomes [27, 29, 31], two blood lipid outcomes [27, 29], and three glucose metabolism outcomes [25, 27, 29]. The findings were mixed. Two studies revealed significant inverse associations between maternal BMI and offspring NCD risk factors [25, 29]. One of these—a cross-sectional study from China—found significant inverse associations between maternal BMI at 15 weeks’ gestation and total cholesterol, LDL cholesterol, 120-min glucose, and 12-min insulin, although it found no significant associations with offspring blood pressure, HDL cholesterol, or fasting glucose or insulin levels [29]. The other—a cross-sectional study from Belgium—revealed significant inverse associations between maternal pre-pregnancy BMI (mppBMI) and proinsulin, β-cell function, fasting insulin (for females only), and insulin resistance (for females only), but not with fasting plasma glucose [25]. Neither of these studies found significant associations between maternal GWG and offspring risk factor outcomes. A cohort study from Israel revealed a significant positive association between mppBMI and offspring blood pressure, although this was attenuated to non-significance after adjusting for adult offspring BMI [27]. The final longitudinal study found no significant relationship between maternal non-pregnant BMI (≥5.5 months post-partum) and offspring blood pressure [31]. There is insufficient evidence from this small number of heterogeneous studies to suggest differences in the effect of maternal BMI by offspring risk factor type. Only one study reported results by sex [25].

### Maternal dietary intake

Ten studies examined the relationship between various aspects of maternal dietary intake on the blood pressure of adult offspring. Five were of high quality, and five of medium quality. The findings were again heterogeneous. Of the five studies that measured levels of maternal protein and carbohydrate intake [31–33, 35, 36], only one—a longitudinal study from Guatemala—found a significant association with offspring NCD risk factors [33]. This study revealed an inverse relationship between prenatal energy intake from supplement and adult fasting plasma glucose, although only in women. However, of the three studies that examined the link between the balance of protein and carbohydrate in the maternal diet and offspring blood pressure, all revealed a significant association [32, 36, 40]. One—a cohort study from Scotland—found that when mothers’ animal protein intake was below 50g/day, an increase in carbohydrate intake was linked with higher offspring blood pressure, while at high daily protein intakes above 50g, greater carbohydrate intake was associated with lower offspring blood pressure [32]. A Dutch cohort study also reported a significant inverse association between the ratio of protein/carbohydrate in mothers’ diets and offspring SBP, in the third trimester specifically [36], while a second Scottish cohort study found that increasing maternal consumption of meat and fish in the context of a high protein-low carbohydrate diet in the second half of pregnancy was linked with significantly higher adult offspring blood pressure [40]. Of the three studies (two RCTs and one cohort study from the same authors in Denmark) that examined maternal gestational fish oil supplementation, none found a significant association with offspring blood pressure [38, 39], blood lipids [37, 39], or glucose metabolism [39]. A single Danish cohort study that measured maternal glycemic index (GI) during pregnancy found a significant positive relationship with offspring total cholesterol and HDL cholesterol (borderline), but not with LDL cholesterol, blood pressure, or glucose metabolism [34]. There was insufficient information from these diverse studies to draw conclusions about differential effects by sex and/or gestational timing of exposure, nor about the influence of adult offspring adiposity.

## Discussion

This systematic review reveals considerable heterogeneity in findings across studies of the impact of various aspects of maternal nutritional status during pregnancy on NCD risk factors in adult offspring. There is evidence of a link between maternal exposure to famine during pregnancy and adverse blood pressure, blood lipid, and glucose metabolism outcomes in adult offspring in some contexts. The evidence base for maternal BMI and GWG is more limited and currently reveals no consistent support for a link between either of these exposures and adult offspring NCD metabolic risk factors. Similarly, there is no indication from the currently limited evidence base of a relationship between the absolute levels of specific maternal dietary nutrients and offspring NCD risk factor outcomes, although there is some tentative support for a possible link between the *balance* of protein and carbohydrate in the maternal diet and offspring blood pressure. This warrants further investigation. There is insufficient evidence from the included papers to assess the influence of offspring sex or gestational timing of exposure. The findings do, however, show some tentative support for an influence of offspring adiposity in the maternal exposure to famine—offspring NCD risk factor relationship, with several studies reporting stronger associations for offspring with high adult adiposity, or a significant attenuation of associations when controlling for this variable.

To our knowledge, this is the first study to systematically review the evidence on the relationship between maternal nutrition during pregnancy and the development of three key NCD metabolic risk factors in adult offspring. Our finding of a link between maternal exposure to famine and offspring NCD risk factors in some contexts is consistent with the findings of existing narrative reviews on the topic [5, 6]. This relationship could be driven by the ‘predictive adaptive response hypothesis’, a form of developmental plasticity in which in utero and early life conditions prompt the development of a phenotype which is adaptive in a ‘predicted’ later life environment [42]. However, where the predicted environment does not match the offspring’s actual later life environment (e.g. where the in utero/early life environment is nutritionally scarce, but the later life environment is nutritionally rich), the phenotype developed (e.g. one suitable for a nutritionally scarce environment) can be maladaptive, leading to deleterious health consequences in adulthood [42]. The mechanisms underlying this plasticity are likely to be multifactorial, but could include alterations in cell number and/or cell type [43, 44], altered maternal hypothalamic-pituitary-adrenal axis activity [45], epigenetic regulation of gene expression [46, 47], and reduced oxidative capacity [48].

The absence of evidence from our included studies of a link between maternal BMI and GWG align with the results of a recent non-systematic review, which found no consistent associations between maternal BMI and CVD risk factors in adults [7]. Our findings in several papers of stronger associations between maternal exposure to famine and offspring NCD risk factors in offspring with high adult adiposity, and the attenuation of associations when controlling for adult adiposity, are consistent with the hypothesis that the impact of development in utero depends strongly on the postnatal environment [42]. Where poor in utero nutrition is combined with later life exposure to obesogenic environments, the effects in terms of offspring’s NCD risk factors and outcomes are likely to be most severe. This is particularly concerning given the rapid nutritional transitions taking place within many developing countries at present.

One key observation to emerge from this systematic review is that of inconsistencies in the results of papers apparently measuring the same maternal nutrition exposure and offspring NCD risk factor. Such mixed findings may stem from differences in sample population, study design including offspring age at follow-up, variation in definitions or measurements, confounding variables, and contextual factors, particularly postnatal environmental life exposures. For the famine studies, for instance, the fact that significant associations were observed for most studies of the Chinese and Dutch famines but not the Leningrad siege might reflect the fact that the former two famines were preceded and followed by sufficient nutrition, whereas the population exposed to the latter was largely malnourished before the siege and remained malnourished for an extended period afterwards [21].

The main strengths of this review lie in its systematic approach and comprehensive scope. Our methodology was designed to capture all studies on a wide range of maternal nutrition exposures and three of the four main NCD metabolic risk factors. To our knowledge, this is true of no other study to date. One consequence of this broad scope, however, is the inclusion of heterogeneous exposure and outcome measures, which limit our ability to synthesise findings. Moreover, the large volume of data returned precludes an in-depth analysis of each risk factor.

In terms of individual studies, the risk of residual confounding is a major source of bias. Important potential confounders include fixed genetic variants common to both mothers and offspring, as well as shared postnatal dietary and lifestyle factors. Whilst ethical considerations restrict the use of strict RCTs to minimise confounding in dietary studies, sibling comparison studies present an opportunity to reduce confounding due to familial/genetic factors. There was considerable loss to follow-up in many cohort studies, a trade-off of the need for long follow-up periods in the assessment of NCD risk factor outcomes. A further source of bias relates to the ascertainment of maternal nutritional exposures. Most existing famine studies lack reliable information about the food intake of individuals during the famine period. Moreover, associations are complicated by the absence of appropriate tools to measure dietary intake accurately in humans.

One of the main contributions of this systematic review is its exposure of the paucity of the current evidence base on the intergenerational links of maternal nutrition during pregnancy and NCD risk factors in adult offspring. Existing studies are drawn disproportionately from high-income countries, particularly in the European Region, which limits the generalisability of the findings. Moreover, while a number of studies have examined the effects of maternal gestational exposure to famine, our search revealed no records investigating the effects of chronic energy deficiency, arguably a more prevalent problem in LMICs today. There are also relatively few studies investigating maternal BMI, GWG, and specific dietary factors, making it difficult to draw clear conclusions about the role of these influences on offspring NCD risk factors. Furthermore, no studies examined the impact of maternal nutrition during lactation. Each of these constitute priority areas for future research, particularly the area of nutrition during lactation given the potential capacity of good nutrition during this time period to offset negative effects of poor nutritional exposure in utero [49]. Future studies that stratify results by sex and gestational timing of exposure and the measurement of postnatal environmental factors will be important, as will research to elucidate the biological mediators and mechanisms underlying the observed associations.

This systematic review reveals considerable heterogeneity in findings across studies. The evidence supports a link between maternal exposure to famine during pregnancy and offspring NCD metabolic risk factors in some contexts, with some tentative support for an influence of adult offspring adiposity in this relationship. Based on an admittedly more limited evidence base, there is no consistent support for relationships between maternal GWG, maternal BMI, or maternal dietary intake and NCD risk factors in adult offspring. Overall, our findings support calls for increased collaboration between maternal nutrition and NCD sectors but suggest that a greater focus on research is needed to identify how these two sectors can work together to support each other’s aims.

## Conclusion and recommendations

Despite calls for increased collaboration and integration of policies and programmes between the maternal nutrition and NCD sectors, there remains weak evidence on the link. Findings of a link between maternal exposure to famine during pregnancy and offspring NCD risk factors in some contexts, plus some evidence of a role of adult adiposity in influencing this relationship, suggest there is potential for significant co-benefits from collaboration. However, more research and evidence are needed to inform how NCD and maternal health sectors can work together to achieve the Sustainable Development Goals (SDGs).

## Supplementary Information

**Additional file 1.** PRISMA checklist.

**Additional file 2.** Search terms.

**Additional file 3.** Quality scoring rubic.

**Additional file 4.** Quality Assessment Scores.

**Additional file 5.** Supplementary Table 1. Findings by maternal nutrition exposure.

## Data Availability

Not applicable.

## Abbreviations

BMI: Body mass index
DALYs: Disability-adjusted life years
GWG: Gestational weight gain
GWG30: Gestational weight gain during the first 30 weeks of gestation
GI: Glycemic index
HDL: High-density lipoproteins
HOMA-IR: Homeostatic model assessment for insulin resistance
LLMIC: Low- and lower middle-income countries
LDL: Low-density lipoprotein
LMIC: Lower middle-income countries
mppBMI: Maternal pre-pregnancy BMI
NCDs: Non-communicable diseases
RCTs: Randomised-controlled trials
SDGs: Sustainable Development Goals
SBP: Systolic blood pressure
WHO: World Health Organization
