# Supplementary material for: Maternal nutrition and its intergenerational links to non-communicable disease metabolic risk factors: a systematic review and narrative synthesis
Source: J Health Popul Nutr. 2021 Apr 26;40:20. doi: 10.1186/s41043-021-00241-2 (PMC8077952; doi:10.1186/s41043-021-00241-2)
Supplement: Supplementary file 2 — Additional file 2. Search terms. [file 41043_2021_241_MOESM2_ESM.docx]

**Search terms**

| # ▲ | Searches | |
| --- | --- | --- |
| 1 | Pregnancy/ | |
| 2 | exp Pregnancy Trimesters/ | |
| 3 | Pregnant Women/ | |
| 4 | preconception care/ or prenatal care/ | |
| 5 | Maternal Health/ | |
| 6 | (pregnant or pregnancy or gestational or preconcept* or pre-concept* or prepregnan* or pre-pregnan*).ti. |  |
| 7 | Postpartum Period/ | |
| 8 | Lactation/ | |
| 9 | Breast Feeding/ | |
| 10 | Bottle Feeding/ | |
| 11 | Infant Food/ | |
| 12 | Weaning/ | |
| 13 | (postnatal or post-natal or postpartum or post-partum).ti,ab. | |
| 14 | (lactation or lactating or breastfeed* or breast feed* or breast fed or breastfed or bottle feed* or bottle fed or bottlefeed or bottlefed or infant feeding or weaning or weaned).ti. | |
| 15 | 1 or 2 or 3 or 4 or 5 or 6 or 7 or 8 or 9 or 10 or 11 or 12 or 13 or 14 or 15 | |
| 16 | Weight Gain/ | |
| 17 | overweight/ or obesity/ or obesity, morbid/ | |
| 18 | Overnutrition/ | |
| 19 | (obes* or overweight or adipos*).ti. | |
| 20 | Weight Loss/ | |
| 21 | Thinness/ | |
| 22 | malnutrition/ or starvation/ | |
| 23 | exp Deficiency Diseases/ | |
| 24 | exp *Diet/ | |
| 25 | exp *Food/ | |
| 26 | Sodium Chloride, Dietary/ | |
| 27 | exp Drinking Behavior/ | |
| 28 | exp *Beverages/ | |
| 29 | (diet or nutrition* or undernutrition or malnutrition or starvation or famine or underweight or thin or slim).ti. | |
| 30 | ((energy or calor* or food or drink*) adj2 (intake or consumption)).ti,ab. | |
| 31 | ((healthy or healthful) adj eating).ti,ab. | |
| 32 | (fruit? or vegetable? or salt or sodium or vitamin? or mineral? or nutrient? or micronutrient?).ti. | |
| 33 | ((fruit? or vegetable? or salt or sodium or vitamin? or mineral? or nutrient? or micronutrient?) adj3 (intake or consum* or diet*)).ti,ab. | |
| 34 | ((vitamin? or mineral? or diet* or nutrition* or nutrient? or micronutrient?) adj3 (deficiency or deficient)).ti,ab. | |
| 35 | (artificial sweetener? or artificial sweetening or sugar substitute? or sweetening agent? or corn syrup? or fructose).ti,ab. | |
| 36 | (alcohol* or drink*).ti. | |
| 37 | (alcohol* adj3 (drink* or factor* or pattern* or habit* or consum* or unhealthy)).ti,ab. | |
| 38 | (((carbonated or sugar* or sweetend or soft) adj2 (drink? or bevarage?)) or soda? or fruit juice?).ti,ab. | |
| 39 | Body Mass Index/ | |
| 40 | (body mass or bmi).ti. | |
| 41 | 16 or 17 or 18 or 19 or 20 or 21 or 22 or 23 or 24 or 25 or 26 or 27 or 28 or 29 or 30 or 31 or 32 or 33 or 34 or 35 or 36 or 37 or 38 or 39 or 40 | |
| 42 | 15 and 41 | |
| 42 | ((maternal or mother*) adj2 (weight or bodyweight or overweight or obes* or adipos* or bmi or body mass or underweight or undernutrition or malutrition or starvation or thin* or slim or excess weight or excess fat)).ti,ab. | |
| 43 | ((pregnant or pregnancy or prepregnan* or pre-pregnan* or gestational) adj2 (weight or bodyweight or overweight or obes* or adipos* or bmi or body mass or underweight or undernutrition or malnutrition or starvation or thin* or slim or excess weight or excess fat)).ti,ab. | |
| 44 | ((preconcept* or pre-concept*) adj2 (weight or bodyweight or overweight or obes* or adipos* or bmi or body mass or underweight or undernutrition or malnutrition or starvation or thin* or slim or excess weight or excess fat)).ti,ab. | |
| 45 | ((prenatal or pre-natal or prepartal or prepartum or pre-partal or pre-partum or antenatal or antepartum or ante-natal or ante-partum) adj2 (weight or bodyweight or overweight or obes* or adipos* or bmi or body mass or underweight or undernutrition or malnutrition or starvation or thin* or slim or excess weight or excess fat)).ti,ab. | |
| 46 | ((maternal or mother*) adj2 (diet* or nutrition*)).ti,ab. | |
| 47 | ((pregnant or pregnancy or prepregnan* or pre-pregnan* or gestational) adj2 (diet* or nutrition*)).ti,ab. | |
| 48 | ((preconcept* or pre-concept*) adj2 (diet* or nutrition*)).ti,ab. | |
| 49 | ((prenatal or pre-natal or prepartal or prepartum or pre-partal or pre-partum or antenatal or antepartum or ante-natal or ante-partum) adj2 (diet* or nutrition*)).ti,ab. | |
| 50 | maternal nutritional physiological phenomena/ or prenatal nutritional physiological phenomena/ | |
| 51 | ((postnatal or post-natal or postpartum or post-partum) adj2 (weight or bodyweight or overweight or obes* or adipos* or bmi or body mass or underweight or undernutrition or malutrition or starvation or thin* or slim or excess weight or excess fat)).ti,ab. | |
| 52 | ((lactation or lactating or breastfeed* or breast feed* or breast fed or breastfed or bottle feed* or bottle fed or bottlefeed or bottlefed or infant feeding or weaning or weaned) adj2 (weight or bodyweight or overweight or obes* or adipos* or bmi or body mass or underweight or undernutrition or malnutrition or starvation or thin* or slim or excess weight or excess fat)).ti,ab. | |
| 53 | ((postnatal or post-natal or postpartum or post-partum) adj2 (diet* or nutrition*)).ti,ab. | |
| 54 | ((lactation or lactating or breastfeed* or breast feed*) adj2 (diet* or nutrition*)).ti,ab. | |
| 55 | Infant Nutritional Physiological Phenomena/ | |
| 56 | 42 or 43 or 44 or 45 or 46 or 47 or 48 or 49 or 50 or 51 or 52 or 53 or 54 or 55 | |
| 57 | exp child/ or infant/ | |
| 58 | (infant? or infancy or child* or preschool* or pre-school* or toddler* or pediatric* or paediatric or juvenile or offspring).ti. | |
| 59 | 57 or 58 | |
| 60 | overweight/ep or obesity, morbid/ep | |
| 61 | exp Body Composition/ | |
| 62 | growth/ or body size/ or body height/ or body weight/ or birth weight/ | |
| 63 | Blood Pressure/ | |
| 64 | Hypertension/ | |
| 65 | Cholesterol/bl [Blood] | |
| 66 | Triglycerides/bl [Blood] | |
| 67 | Lipids/bl [Blood] | |
| 68 | exp Hyperlipidemias/ | |
| 69 | Blood Glucose/ | |
| 70 | Insulin/bl [Blood] | |
| 71 | Puberty, Precocious/ | |
| 72 | Mortality/ | |
| 73 | Asthma/ | |
| 74 | exp Pulmonary Disease, Chronic Obstructive/ | |
| 75 | exp *Neoplasms/ | |
| 76 | (obes* or overweight* or bmi or body mass).ti. | |
| 77 | (growth or height or body composition or body fat or body weight or bodyweight or weight gain or muscle mass).ti. | |
| 78 | (blood pressure or hypertens*).ti. | |
| 79 | ((blood or plasma or serum) adj2 (lipid* or cholesterol or triglyceride*)).ti,ab. | |
| 80 | (hypercholesterol?emi* or hyperlipid?emi?).ti. | |
| 81 | (lipid* or cholesterol or triglyceride*).ti. | |
| 82 | ((blood or plasma or serum) adj2 glucose).ti,ab. | |
| 83 | (insulin adj2 (sensitivity or tolerance or growth factor)).ti,ab. | |
| 84 | (asthma* or ((lung or respiratory or pulmonary) adj function)).ti,ab. | |
| 85 | (chronic adj2 (lung oe pulmonary or bronchitis)).ti,ab. | |
| 86 | (cancer? or neoplas* or carcinoma? or leukaemia? or leukemia? or lymphoma? or sarcoma? or malignanc*).ti. | |
| 87 | (mortality or death?).ti. | |
| 88 | 60 or 61 or 62 or 63 or 64 or 65 or 66 or 67 or 68 or 69 or 70 or 71 or 72 or 73 or 74 or 75 or 76 or 77 or 78 or 79 or 80 or 81 or 82 or 83 or 84 or 85 or 86 or 87 | |
| 89 | 56 and 59 and 88 | |
| 90 | Pediatric Obesity/ | |
| 91 | Child Mortality/ or Infant Mortality/ | |
| 92 | Prenatal Exposure Delayed Effects/ | |
| 93 | ((infant? or infancy or child* or preschool* or pre-school* or toddler* or pediatric* or paediatric or juvenile or offspring) adj2 (obes* or overweight)).ti,ab. | |
| 94 | ((infant? or infancy or child* or preschool* or pre-school* or toddler* or pediatric* or paediatric or juvenile or offspring) adj5 (lipid* or cholesterol or triglyceride* or hypercholesterol?emi* or hyperlipid?emi?)).ti,ab. | |
| 95 | ((infant? or infancy or child* or preschool* or pre-school* or toddler* or pediatric* or paediatric or juvenile or offspring) adj5 (blood glucose or insulin)).ti,ab. | |
| 96 | ((infant? or infancy or child* or preschool* or pre-school* or toddler* or pediatric* or paediatric or juvenile or offspring) adj5 (blood pressure or hypertens*)).ti,ab. | |
| 97 | ((infant? or infancy or child* or preschool* or pre-school* or toddler* or pediatric* or paediatric or juvenile or offspring) adj5 (growth or height or body composition or body fat or body weight or bodyweight or weight gain or muscle mass)).ti,ab. | |
| 98 | ((postnatal or post-natal or postpart* or post-part* or "catch up") adj2 (growth or weight gain)).ti,ab. | |
| 99 | ((early or precocious or premature) adj2 puberty).ti,ab. | |
| 100 | ((infant? or infancy or child* or preschool* or pre-school* or toddler* or pediatric* or paediatric or juvenile or offspring) adj2 (mortality or death?)).ti,ab. | |
| 101 | ((infant? or infancy or child* or preschool* or pre-school* or toddler* or pediatric* or paediatric or juvenile or offspring) adj5 (cancer? or neoplas* or carcinoma? or leukaemia? or leukemia? or lymphoma? or sarcoma? or malignanc*)).ti,ab. | |
| 102 | 90 or 91 or 92 or 93 or 94 or 95 or 96 or 97 or 98 or 99 or 100 or 101 | |
| 103 | 42 and 102 | |
| 105 | exp animals/ not humans.sh. | |
| 106 | (rat or rats or mouse or mice or murine or rodent?).ti. | |
| 107 | 105 and 106 | |
| 108 | 103 not 107 | |
