# Supplementary material for: Maternal nutrition and its intergenerational links to non-communicable disease metabolic risk factors: a systematic review and narrative synthesis
Source: J Health Popul Nutr. 2021 Apr 26;40:20. doi: 10.1186/s41043-021-00241-2 (PMC8077952; doi:10.1186/s41043-021-00241-2)
Supplement: Supplementary file 3 — Additional file 3. Quality scoring rubic. [file 41043_2021_241_MOESM3_ESM.docx]

**Quality scoring rubric**

| **Study type** | 1 | 2 | 3 | 4 | 5 | 6 | 7 | 8 | **Total score**  0  1  2 |
| --- | --- | --- | --- | --- | --- | --- | --- | --- | --- |
| **Case-Control** | Is Case Definition Adequate? | Representativeness of the Cases | Selection of Controls | Definition of Controls | Comparability of cases and controls on basis of design/analysis | Ascertainment of Exposure | Non-Response Rate |  |  |
| **Cohort** | Representativeness of the exposed cohort | Selection of the non-exposed cohort | Ascertainment of exposure | Demonstration that outcome of interest was not present at start of study | Comparability of cohorts on the basis of the design or analysis | Assessment of outcome | Was follow-up long enough for outcomes to occur | Adequacy of follow-up of cohorts |  |
| **Cross-sectional** | Representativeness of the sample | Sample Size | Non-respondents | Ascertainment of the exposure | Comparability of subjects in different outcome groups (control for confounding) | Assessment of the outcome | Statistical test is appropriate |  |  |
| **Interrupted time series** | shape of intervention prespecified (they say what they expect to happen) | Intervention independent of other changes/confounders/historic changes | Intervention did not affect data collection/data collection method same pre and post | Allocation concealment (blind or objective outcome assessment) | Incomplete data adequately addressed | all outcomes mentioned in methods are reported in results | free from other sources of bias e.g. seasonality |  |  |
| **RCT** | Random sequence generation (selection bias) | Allocation concealment (selection bias) | Blinding of participants and personell (performance bias) | Blinding of outcome assessment (detection bias) | Incomplete oucome data (reporting bias) | Selective reporting (reporting bias) | Other bias |  |  |

Score 1=achieved, 0=not achieved, 2=unclear [RCT only]
