# Supplementary material for: Maternal nutrition and its intergenerational links to non-communicable disease metabolic risk factors: a systematic review and narrative synthesis
Source: J Health Popul Nutr. 2021 Apr 26;40:20. doi: 10.1186/s41043-021-00241-2 (PMC8077952; doi:10.1186/s41043-021-00241-2)
Supplement: Supplementary file 4 — Additional file 4. Quality Assessment Scores. [file 41043_2021_241_MOESM4_ESM.docx]

Quality Assessment

**Cohort studies**

| Reference (year). Country. | Representativeness of the exposed cohort | Selection of the non-exposed cohort | Ascertainment of exposure | Demonstration that outcome of interest was not present at start of study | Comparability of cohorts on the basis of the design or analysis | Assessment of outcome | Was follow-up long enough for outcomes to occur | Adequacy of follow-up of cohorts | Additional Bias |
| --- | --- | --- | --- | --- | --- | --- | --- | --- | --- |
| Campbell et al. (1996), UK | B | A* | C | A* | B* | A* | A* | B* |  |
|  | Only primiparous women who were married and who took part in a survey of diet in late pregnancy and had remained in the area where they were born | Controls from same Aberdeen Maternity Hospital | Food diaries completed by mothers | Followed from birth | Controlled for sex, BMI, alcohol consumption, cuff size, though not other adult lifestyle factors | Clinical examination | 18+ years | Studied 76% of the 331 people whom we were able to trace (>50%) |  |
| Conlisk et al (2004), Guatemala | B | B* | A* | A* | B* | A* | A* | B* |  |
|  | Dependent upon attendance at village feeding hall, which varied by village from 65-85%. | From matched villages randomised to receive a different supplement | Recorded supplement intake (to the nearest 10ml). | Followed from birth | Controlled for SES, gestational age, age, energy intake, physical activity and migration (also controlled for adult BMI although results similar to those without controlling for BMI so these not shown) | Fasting finger prick blood glucose test | 18+ years | Examined 78% of those who could be traced with exclusions explained (>50%) |  |
| Danielsen et al (2013), Denmark | B | A* | A* | A* | B* | A* | A* | C |  |
|  | Dependent upon agreement and completion of questionnaire by eligible mothers | Controls from same population | Food frequency questionnaire and structured interview | Followed from birth | Adjusted for maternal smoking, BMI, education, energy intake and offspring’s physical activity level but not offspring’s dietary intake | Clinical examination | 18+ years | 44% of original cohort (<50%), 49% of traced individuals (<50%) | Potential non-response bias – mothers of participating offspring more often normal weight and non-smokers and had higher educational attainment than mothers of non-participating offspring. |
| De Rooij et al (2007), Netherlands | B* | B* | B | A* | B* | A* | A* | B* | Potential selective participation if those who were fit enough were more likely to attend clinical at 58y. |
|  | Comprehensive recruitment but follow up relied on living in the same area 58 years later | Born in same location, different time period | Assumed by birth location, time and historical records. | Followed from birth | Adjusted for sex and BMI, though not for offspring’s behavioural risk factors | Clinical examination | 18+ years | 55% of eligible cohort (>50%) |  |
| Hochner et al (2012), Israel | A* | A* | B* | A* | B* | A* | A* | A* |  |
|  | Population-based cohort | Non-exposed drawn from the same community as exposed cohort | mppBMI and GWG reported by mothers in interviews conducted by nurses while hospitalised after delivery. | Followed from birth | Controlled for ethnicity, sex, parity, mother's age, maternal smoking, maternal SES, maternal years of education, maternal medical condition, birth weight, gestational week, and offspring characteristics at 32 years of age (smoking status, physical activity, years of education), but not adult BMI? | Clinical examination | 18+ years | All subjects accounted for? |  |
| Hrolfsdottir et al (2015), Denmark | B | A* | C | A* | A* | A* | A* | C |  |
|  | Lower energy intake, lower educational level and higher prevalence of primi- and multiparity in mothers attending vs. not attending clinical examination | Non-exposed drawn from the same community as exposed cohort | Maternal pre-pregnancy weight was based on self-report | Followed from birth | Controlled for maternal pre-pregnancy BMI, age, parity, smoking status, educational level, (offspring's sex), whether offspring thinks their father is overweight, plus additional adjustment for offspring smoking, alcohol habits and BMI at age 20 | Clinical examination | 18+ years | Studied 45% of the follow-up cohort (<50%) |  |
| Huang et al (2010), China | C | B* | B | A* | - | A* | A* | ? |  |
|  | Women only | Same community, different time period | Assumed by birth location, time and historical records. | Followed from birth | No mention of control for any factors | Clinical examination | 18+ years |  |  |
| Mamun et al (2009), Australia | A* | A* | C | A* | B* | A* | A* | B* | Large loss to follow-up – resulting in limited statistical power |
|  | Population-based sample from major public hospital | Controls drawn from same population-based hospital sample | Gestational weight gain during pregnancy calculated from measured maximum weight in pregnancy and self-reported prepregnancy weight | Followed from birth | Controlled for maternal age at birth, maternal education, parity, cigarette smoking, maternal ppBMI but did not control for offspring BMI or behavioural risk factors. | Clinical examination | 18+ years | Studied 53% of eligible cohort, (>50%) |  |
| De Rooij et al (2006), Netherlands | B* | B* | B | A* | B* | A* | A* | C |  |
|  | Comprehensive recruitment but follow up relied upon living in the same area 58 years later | Born in same location, different time period | Assumed by birth location, time and historical records. | Followed from birth | Adjusted for sex, BMI. | Clinical examination and linked records | 18+ years | Studied 29% of original cohort (<50%) |  |
|  | A* | A* | B | A* | B* | A* | A* | C |  |
| Ravelli et al (1998), Netherlands | Studied population matched to total population born in the area | Random sample from same hospital taken before and after famine | Assumed by birth location, time and historical records. | Followed from birth | Adjusted for sex and adult BMI, not for adult behavioural factors. | Linked records and medically accredited IGTT | 18+ years | Only 702 (13%) subjects studied of an identified 5425 potential participants (<50%) | Calorie intake unknown (other sources of food available on top of official rations). |
| Roseboom et al (1999), Netherlands | B* | B* | B | A* | A* | A* | A* | C |  |
|  | Comprehensive recruitment but follow up relied upon living in the same area ˜50 years later | Born in same location, different time period | Assumed by birth location, time and historical records. | Followed from birth | Controlled for age, sex, maternal characteristics (weight at end of pregnancy and weight gain), adult characteristics (BMI, SES, use of anti-hypertensive medication). | Clinical examination | 18+ years | 34% of traced individuals followed up (<50%) |  |
|  | B* | B* | B | A* | A* | A* | A* | C |  |
| Roseboom et al (2000), Netherlands | Comprehensive recruitment but follow up relied upon living in the same area ˜50 years later | Same hospital community, although different time of birth | Assumed by birth location, time and historical records. | Followed from birth | Controlled for sex, adult BMI, adult SES, smoking status, use of lipid lowering medication, maternal (age, parity, weight at last prenatal visit, SES), infant feeding practice | Clinical examination | 18+ years | 33% of traced individuals followed up (<50%) |  |
|  | B* | A* | B | A* | A* | A* | A* | C |  |
| Roseboom et al (2001), Netherlands | Babies born in Wilhelmina Gasthuis university hospital, but needed to live in or close to Amsterdam to be included in follow up | Different exposure groups drawn from same community | Assumed by birth location, time and historical records. | Followed from birth | Adjusted for sex, plus adult BMI, smoking, SES, and age. Also adjusted for maternal weight gain and weight at end of pregnancy and size of baby at birth. | Clinical examination | 18+ years | 30% of traced individuals (<50%) |  |
|  | A* | A* | B | A* | B* | A* | A* | B* |  |
| Rytter et al (2013), Denmark | Mothers attending routine antenatal care at medical centre in Aarhus medical centre, Denmark | Controls drawn from same medical centre | Structured interview plus food diary | Followed from birth | Controlled for maternal pre-pregnancy BMI, maternal education, smoking during pregnancy, maternal age, parity, energy intake and sex; no mention of control for offspring lifestyle risk factors or adult BMI | Clinical examination | 18+ years | 46% of original cohort (just <50%) but description provided of those lost and participation was not associated with exposure in the study | Loss to follow up – limited power; self-reported food diary – potentially imprecise estimates of exposure |
|  | B* | A* | C | A* | B | A* | A* | B* |  |
| Shiell et al (2001), UK | Study confined to persons born in Motherwell Maternity Hospital who still lived in the area 29 years later. | All participants drawn from same community, gradient of intake of various nutrients so no non-exposed cohort as such. | Reported dietary intake in specially designed form | Followed from birth | Did not control for maternal intake of saturated fat, salt etc. | Clinical examination | 18+ years | 65% of traced individuals followed up (>50%) | Crude measures of maternal dietary exposure variable; possible confounding by high saturated fat/salt and/or reduced GWG |
|  | B* | B* | B | A* | A* | A* | A* | C |  |
| Stein et al (2006), Netherlands | Comprehensive recruitment from midwifery training schools and university hospital but individuals born out of wedlock were not traced | Same study hospitals or same-sex siblings, different time period | Assumed by birth location, time and historical records. | Followed from birth | Controlled for age, sex smoking, alcohol intake, height, waist circumference | Clinical examination | 18+ years | Studied 42% of traced individuals (<50%) |  |
|  | B* | B* | A* | A* | A* | A* | A* | B* |  |
| Webb et al (2005), Guatemala | Eligible participants different from eligible non-participants and from ineligible members of the birth cohort with respect to birth weight, supplement group, and maternal supplement intake in the Atole villages, plus relied on participants still living in same area at follow up | From matched villages randomised to receive a different supplement | Recorded supplement intake (to the nearest 10ml) | Followed from birth | Controlled for sex, BMI, WHR, SES at birth, current residence (urban/rural), attained education, current PA level, age and follow-up, smoking status (yes/no) and alcohol consumption (yes/no) | Clinical examination | 18+ years | 77% of eligible cohort measures examined (>50%) | Not able to separate effects of protein and energy supplementation. Participation bias: significant differences in birth weight, supplement group, and maternal supplement intake in the Atole villages were observed between participants and non-participants |

**Cross-sectional studies**

| Reference (year). Country. | Representativeness of the sample | Sample Size | Ascertainment of Exposure | Non-respondents | Comparability of outcome groups | Assessment of outcome | Statistical test | Adequacy of follow-up period | Additional Bias |
| --- | --- | --- | --- | --- | --- | --- | --- | --- | --- |
| Li et al (2010), China | A* | A* | B |  | B* | A* | A* | A* |  |
|  | Data from China National Nutritional and Health Survey (CNNHS), a nationally representative cross-sectional study | N of interest =2959 (total N=7874) | Exposure to famine based on year of birth | No description of response rate | Controlled for sex, family history of diabetes, educational level, current smoking, alcohol use and physical activity level, but not adult BMI | Clinical examination | Yes | 18+ years | Assumed that residents investigated were born in the same province and in a similar rural area.  Lack of reliable information about individual food availability during the famine. |
| Loos et al (2002), Belgium | A* | A* | B* | A* | A* | A* | A* | A* |  |
|  | Population based sample. | N=800 individual twins, satisfactory | Self-reported weight before pregnancy and weight gain | 52.7% (>50%) | Controlled for age, sex, gestational age, BMI. | Clinical examination | Yes | 18+ years |  |
| Mi et al (2000), China | B* | A* | A* | B | A* | A* | A* | A* |  |
|  | Offspring born in the Peking Union Medical College Hospital in Beijing. Relied on mothers still living in Beijing about 45 years after birth | N=627, satisfactory | Clinical examination of maternal height and weight by blinded observers | Traced and examined only 33% of eligible adult offspring (<50%) | Adjusted for sex and BMI | Clinical examination | Yes | 18+ years |  |
| Scheers-Andersson et al (2015), Sweden | C | A* | A* | A* | B* | B* | A* | A* | Analysis on sibling pairs may suffer from too low statistical power |
|  | Only includes women with at least two male children (full brothers) | N=9816 brothers, satisfactory | Clinical measurement | No non-respondents (data from publically accessible military conscription records) | Controlled for genetic effects (sibling-pair study). Controlled for maternal age at birth, birth year, gestational age, maternal early-pregnancy BMI, parity and maternal education, although not for offspring BMI | Record linkage | Yes | 18+ years |  |
| Stanner et al (1997), Russia | B* | A* | B | B | B* | A* | A* | A* |  |
|  | Relied on participants living in same area ˜50 years later | Sample size = 549 (169 exposed), satisfactory | Exposure to famine based on year of birth | Low response rate (28% male, 58% female) | Controlled for sex, but not adult NCD risk factors e.g. BMI | Clinical examination | Yes | 18+ years |  |
| Wang et al (2015), China | A* | A* | B | A* | B* | A* | A* | A* | Lack of reliable information about individual food availability during the famine.  Actual calorie intake unknown (other sources of food available on top of official rations). |
|  | Population-based sample obtained using stratified cluster sampling method | N of interest =2420 (total N=6445), satisfactory | Exposure to famine based on year of birth | High response rate (90.8%) | Controlled for age, but not adult NCD risk factors e.g. BMI | Clinical examination | Yes | 18+ years |  |
| Zheng et al (2012), China | C | A* | B | C | B* | A* | A* | A* |  |
|  | Volunteers and urban residents only | Sample size = 5040, satisfactory | Exposure to famine based on year of birth | No description of response rate | Controlled for age, but not adult NCD risk factors e.g. BMI | Clinical examination | Yes | 18+ years |  |

**RCTs**

| Reference (year). Country. | Random Sequence generation | Allocation concealment | Blinding of participants and personnel | Blinding of Outcome Assessment | Incomplete outcome data | Selective reporting | Additional bias |
| --- | --- | --- | --- | --- | --- | --- | --- |
| Macleod et al (2013), UK | A* | B | B | A* | A* | A* |  |
|  | Yes | No | No | Yes | Analysed 50% of traced offspring. | Limited risk of selected reporting |  |
| Rytter et al. (2011), Denmark | A* | A* | A* | A* | B | A* | Loss to follow-up and consequent limited power |
|  | Yes | Yes | Identical looking capsules in identical boxes (OO - reference group vs. FO); no oil group was unblinded although this was not the reference group | No mention (although used objective clinical examination to detect outcomes) | Larger loss to follow up for FO (41% included) vs. OO (53% included) and NO (48% included) | Limited risk of selected reporting |  |
| Rytter et al. (2012),  Denmark | A* | A* | A* | A* | B | A* | Loss to follow-up and resulting limited power |
|  | *Assuming same as Rytter et al 2011 | *Assuming same as Rytter et al 2011 | Identical looking capsules in identical boxes (OO - reference group vs. FO); no oil group was unblinded although this was not the reference group | All outcomes were clinically measured by assistants who were blinded for study allocation | Larger loss to follow up for FO (41% included) vs. OO (53% included) | Limited risk of selected reporting |  |
