# Supplementary material for: Maternal nutrition and its intergenerational links to non-communicable disease metabolic risk factors: a systematic review and narrative synthesis
Source: J Health Popul Nutr. 2021 Apr 26;40:20. doi: 10.1186/s41043-021-00241-2 (PMC8077952; doi:10.1186/s41043-021-00241-2)
Supplement: Supplementary file 5 — Additional file 5. Supplementary Table 1. Findings by maternal nutrition exposure. [file 41043_2021_241_MOESM5_ESM.docx]

**Supplementary Table 1: Findings by exposure**

**Famine**

| **Authors (year), country, study design** | **Sample description** | **Exposure** | **Outcome measures** | **Outcomes** |
| --- | --- | --- | --- | --- |
| Stanner et al (1997)  Russia  Cross-sectional | N=549, 27% male, aged 52-53 years  Three groups: intrauterine group: 169 subjects exposed to malnutrition in utero;  Infant group: 192 subjects exposed to malnutrition in infancy but not in utero;  Unexposed group: 188 subjects born concurrently with other 2 groups but outside area of siege | Exposure to famine in utero (Leningrad siege) | SBP (mmHg)  DBP (mmHg)  Total cholesterol (mmol/L)  LDL (mmol/L)  HDL (mmol/L)  Triglyceride (mmol/L)  Plasma glucose (fasting, 30min, 120min) (mmol/L)  Impaired glucose tolerance (%)  Plasma insulin (fasting, 30min, 120min) (mmol/L)  Fasting plasma proinsulin  Fasting plasma des 31,32 proinsulin (pmol/L)  Proinsulin-like molecules (%) | No significant difference in SBP between groups (131.6 vs. 133.1 vs. 128.5, p>0.05).  DBP marginally lower in unexposed group compared to exposed groups (80.9 vs. 82.2 vs 77.3, 0.005<p<0.05).  No significant difference in any blood lipid concentrations between groups (p>0.05).  No significant difference in any glucose level/metabolism outcome between groups (p>0.05).  *Adjusted for sex |
| Zheng et al (2012) [19]  China  Cross-sectional | N of interest=3696, total N=5040, 62% male, aged 44-51 years  Urban residents from Chongqing, who volunteered to participate in the annual routine physical examinations (Jan-Dec 2008), born around the great Chinese famine (1957-1964). Three groups based on year of birth: fetally exposed, postnatally exposed, control. | Exposure to famine in utero (Chinese Famine | SBP (mmHg)  DBP (mmHg)  Hypertension (%)  Total cholesterol (mmol/L)  LDL cholesterol (mmol/L)  HDL cholesterol (mmol/L)  Triglyceride (TG) (mmol/L)  Hypertriglyceridemia (triglyceride ≥1.70mmol/L or drug treatment for elevated triglyceride) (%)  Fasting plasma glucose (mmol/L)  Dysglycemia (fasting plasma glucose ≥6.1mmol/L or drug treatment for diabetes) (%) | Significantly higher SBP in fetally exposed vs. non-exposed men (126.7 vs. 125.1, p<0.01) and women (121.8 vs. 117.6, p<0.01); in women, significantly higher DBP (76.8 vs. 75.3, p<0.05) and prevalence of hypertension (16.5% vs. 13.5%, p<0.05), but not in men (p>0.05).  Significantly higher total cholesterol (5.0 vs. 4.9, p<0.05), LDL cholesterol (2.9 vs. 2.8, p<0.05) and prevalence of hypertriglyceridemia (20.2% vs. 16.8%, p<0.05) in fetally exposed vs. non-exposed women, but no significant difference for men (p>0.05); no significant difference in HDL cholesterol or TG for men or women (p>0.05).  Significantly higher fasting plasma glucose (men: 5.5 vs 5.3, p<0.01, women: 5.1 vs. 5.0, p<0.01) and prevalence of dysglycemia (men: 15.6% vs. 10.7%, p<0.05, women: 5.6% vs. 3.3%, p<0.05) in fetally exposed vs. non-exposed.  *Adjusted for age |
| Wang et al (2015)  China  Cross-sectional | N of interest=2420, total N=6445, 42% male, aged 52-53 years  Participants in SPECT-China population-based cross-sectional study, East China. Analysed two groups based on date of birth: fetally exposed and unexposed. | Exposure to famine in utero (Chinese Famine) | SBP (mmHg)  DBP (mmHg)  Hypertension (%)  HDL (mmol/L)  Triglycerides (TG) (mmol/L)  Raised triglycerides (TG) (%)  Fasting plasma glucose (mmol/L)  Homeostatic model assessment -insulin resistance (HOMA-IR) | Significantly higher SBP, DBP and % hypertension in exposed vs. unexposed in women (130 vs. 122, p<0.05, 79 vs. 75, p<0.05, 55.9 vs. 33.4, p<0.05) but not in men (130 vs. 128 p>0.05, 82 vs. 82, p>0.05, 59.5 vs. 53, p>0.05).  Significantly higher prevalence of raised TG in exposed vs. unexposed in women (34.4 vs. 17.8, p<0.05) but not men (48.2 vs. 48.0, p>0.05).  No significant difference in HDL-c between exposed vs. unexposed in either sex.  Significantly higher fasting plasma glucose in exposed vs. unexposed in men (5.9 vs. 5.6, p<0.05) but not in women (5.7 vs. 5.4, p>0.05); significantly higher HOMA-IR in exposed vs. unexposed in women (1.68 vs. 1.42, p<0.05) but not men (1.41 vs. 1.38, p<0.05).  *Adjusted for age |
| Huang et al (2010)  China  Cohort | N of interest=33247, total N=35025, 0% male, aged ˜32 years  Data from 1993-1996 – China-US Collaborative Project for Neural Tube Defect Prevention. 35025 women born in China 1957-1963. Includes cohorts born before (1957, 1958), during (1959, 1961), and after (1962,1963) the famine. | Exposure to famine in utero (Chinese Famine) | Hypertension (%) (=SBP≥140mmHg, DBP≥90mmHg) | No significant difference in prevalence of hypertension in women born during famine (1959, 1960, 1961, 1962) vs. 1963 after famine (p>0.05) in urban or rural samples. |
| Roseboom et al (1999)  Netherlands  Cohort | N=739 (297 exposed in utero). 48% male, aged approx. 51-55 years  Live singletons born at term in the Wilhelmina Gasthuis Hospital in Amsterdam between 1^st^ November 1943 and 28^th^ February 1947. Three exposure groups based on year of birth: early gestation (19^th^ August 1945-8^th^ December 1945), mid gestation (29^th^ April 1945-18^th^ August 1945), late gestation (7^th^ January 1945-28^th^ April 1945) plus two non-exposed groups: born before famine, conceived after famine. | Exposure to famine in utero (Dutch Famine) | SBP (mmHg)  DBP (mmHg) | No significant association between prenatal exposure to famine and SBP or DBP in any of the three exposure groups (p>0.05).  *Adjusted for age, sex, maternal characteristics (weight at end of pregnancy and weight gain), adult characteristics (BMI, SES, use of anti-hypertensive medication). |
| Stein et al (2006)[20]  Netherlands  Cohort | N=971, 45% male, 56-62 years  Exposed = live singleton births at three institutions in famine-exposed cities in 1945 and early 1946; controls = unexposed births in the three hospitals plus the siblings of the exposed birth series | Exposure to famine in utero (Dutch Famine) – defined as exposure to official ration of <900kcal/day for at one 10-week period | SBP (mmHg)  DBP (mmHg)  Hypertension (%) | Significantly higher SBP (β: 2.77, 95% CI: 0.25-5.30), DBP (β: 1.27, 95% CI: -0.13-2.66), and risk of hypertension (OR: 1.44, 95% CI: 1.04-2.00) in fetally exposed vs. non-exposed offspring (adjusted for age and sex), but effect sizes attenuated to null following further adjustment for several adult covariates, most notably waist circumference (SBP: β: 1.95, 95% CI: -0.55-4.45, DBP: β = 0.75, 95% CI: -0.62-2.11, hypertension: OR: 1.32, 95% CI: 0.94-1.84). |
| De Rooij et al (2006)  Netherlands  Cohort | N=672-697, ≈46% male, 58 years  Offspring born between 1943-1947 at Wilhelmina Gasthuis hospital, Amsterdam. Two groups based on date of birth: fetally exposed (inc. early, mid, late gestation), unexposed (born before or conceived after famine). Traced using the national population registry. | Exposure to famine in utero (Dutch Famine) | Fasting plasma glucose (mmol/L), insulin, HbA_1C_(%)  120 min plasma glucose (mmol/L), insulin, proinsulin (pmol/L), 32-33 proinsulin (pmol/L) | Significantly higher 120-min plasma glucose concentrations (difference=0.4, 95% CI: 0.1-0.7, p=0.02) and 120-min plasma insulin concentrations (difference=27, 95% CI: 0-58) in exposed vs. unexposed; no significant associations between exposure to famine in utero and fasting plasma glucose, insulin or HbA_1C_ (p>0.05).  *Adjusted for sex and BMI. |
| De Rooij et al (2007)  Netherlands  Cohort | N=783, 46% male, mean age=58 years  Live singletons born at term in the Wilhelmina Gasthuis Hospital in Amsterdam between 1^st^ November 1943 and 28^th^ February 1947. Three exposure groups based on year of birth: early gestation (19^th^ August 1945-8^th^ December 1945), mid gestation (29^th^ April 1945-18^th^ August 1945), late gestation (7^th^ January 1945-28^th^ April 1945) plus two non-exposed groups: born before famine, conceived after famine. | Exposure to famine in utero (Dutch Famine) | SBP (mmHg)  DBP (mmHg)  HDL cholesterol (mmol/L)  Triacylglycerol (g/L)  Fasting glucose (mmol/L) | No significant difference in SBP (p=0.99) or DBP (p=0.67) for offspring exposed to famine in utero (early, mid or late) versus non-exposed offspring.  Significantly lower HDL cholesterol in exposed versus non-exposed men (difference = -0.008, 95% CI:- 0.14, 0.00, p=0.05), with the greatest difference for exposure in early gestation (-0.13, 95% CI: -0.24, -0.01, p<0.05); trend towards lower HDL cholesterol in exposed versus non-exposed women, although not quite significant (-0.07, 95% CI: -0.14, -0.00, p=0.07); significantly higher triacylglycerol concentrations for exposed versus non-exposed individuals (difference=0.1, 95% CI: 0.0, 0.2, p=0.04).  No significant difference in fasting glucose (p=0.48) for offspring exposed to famine in utero (early, mid or late) versus non-exposed offspring.  *Adjusted for sex and BMI. |
| Roseboom et al (2000)[50]  Netherlands  Cohort | N=704, 48% male, ≈50 years.  Live singletons born at term in the Wilhelmina Gasthuis Hospital in Amsterdam between 1st November 1943 and 28th February 1947. Three exposure groups based on year of birth: early gestation (19th August 1945-8th December 1945), mid gestation (29th April 1945-18th August 1945), late gestation (7th January 1945-28th April 1945) plus two non-exposed groups: born before famine, conceived after famine. | Exposure to famine in utero (Dutch Famine), defined as official daily rations for persons over 21y during any 13-week period of gestation <1000kcal/day. | Total cholesterol (mmol/L)  LDL cholesterol (mmol/L)  HDL cholesterol (mmol/L)  LDL:HDL cjp;esterp;  Apolipoprotein A-I (Apo-AI) (g/L)  Apolipoprotein B (Apo-B) (g/L) | Significantly higher LDL:HDL (13.9% difference, 95% CI: 2.6-26.3, p<0.05), lower (though not significantly) HDL (-7.0 difference, 95% CI: -13.0--0.6) and Apo-A levels (-0.07% difference, 95% CI: -0.14,-0.01), and higher (though not significantly) LDL (0.24% difference, 95% CI:-0.02, 0.52) and Apo-B (0.03% difference, 95% CI:-0.04,0.11) in offspring exposed in early gestation vs. non-exposed; exposure in mid or late gestation associated with lower total and LDL cholesterol levels though differences not significant.  *Results shown are adjusted for sex, but results also independent of size at birth, adult BMI, gestational age at birth, infant feeding practice and adult characteristics e.g. SES, smoking status, use of lipid lowering medication. |
| Ravelli et al (1998)  Netherlands  Cohort | N=702, 48% male, aged 51-55 years  Offspring born between 1943-1947 at Wilhelmina Gasthuis hospital, Amsterdam. Two groups based on date of birth: fetally exposed (inc. early, mid, late gestation), unexposed (born before or conceived after famine). Traced using the national population registry. | Exposure to famine in utero (Dutch Famine) | Fasting glucose (mmol/L), insulin, proinsulin (pmol/L), 32-33 proinsulin (pmol/L)  30 min glucose (mmol/L), insulin, proinsulin (pmol/L), 32-33 proinsulin (pmol/L)  120 min glucose (mmol/L), insulin, proinsulin (pmol/L), 32-33 proinsulin (pmol/L) | Significantly higher 120 min glucose (6.2 vs. 5.8, p=0.006), 120 min insulin (198 vs. 171, p=0.04) and fasting proinsulin (6.2 vs. 5.9, p=0.05) in exposed vs. unexposed.  No significant difference in fasting or 30 min glucose, insulin, proinsulin, 32-33 proinsulin, or relative insulin increment in exposed vs. unexposed, p>0.05.  *Adjusted for sex and BMI. |
| Li et al (2010)  China  Cross-sectional | N of interest=2959 (1005 exposed), total N=7874, 46% male aged 43-45 years  Residents of rural areas, born between 1^st^ October 1952-30^th^ September 1964.  Analysed five groups based on birth date: non-exposed, fetal-exposed, early childhood-exposed, mid childhood-exposed, late childhood-exposed.  Data from the 2002 China National Nutritional and Health Survey (CNNHS). | Exposure to famine in utero (Chinese Famine) | Fasting plasma glucose (mmol/L)  Hyperglycemia (fasting plasma glucose ≥6.1mmol/L and/or 2-h plasma glucose ≥7.8mmol/L and/or a previous diagnosis of type 2 diabetes | Significantly higher mean fasting plasma glucose concentration (4.95 vs. 4.75, p=0.007) and prevalence of hyperglycemia (OR: 3.92, 95% CI: 1.64-9.39) in fetal-exposed vs. non-exposed in severely affected famine areas, but not in less severely affected famine areas (mean FPG: 4.73 vs. 4.81, p=0.234, hypertension OR: 0.57, 95% CI: 0.25-1.31). Significant interaction between famine severity and fetal-exposed vs. non-exposed cohort (p<0.0001 for FPG, p=0.001 for hypertension). Substantially elevated odds of hypertension in fetal-exposed cohort who consumed an affluent/Western diet in adulthood (OR: 7.63, 95% CI: 2.41-24.1, p=0.0005, compared to OR: 2.34, 95% CI: 0.82-6.70, p=0.112 for those with a traditional Chinese dietary pattern), and also in fetal-exposed cohort with higher adult SES (OR: 6.20, 95% CI: 2.08-18.5, p=0.001, compared to 1.68, 95% CI: 0.50-5.71, p=0.404 for subjects with lower adult SES). No significant difference in relative risk of hyperglycemia for overweight fetal-exposed subjects vs. normal weight fetal-exposed subjects.  *Adjusted for sex, family history of diabetes, educational level, current smoking, alcohol use and physical activity level. |

**GWG**

| Mamun et al (2009)  Australia  Cohort | N=2271, 50% male, 21 years  Offspring of women who received antenatal care at a major public hospital in Brisbane (Australia) between 1981-1983, Mater-University Study of Pregnancy and its Outcomes (MUSP). | Maternal GWG | SBP (mmHg)  DBP (mmHg) | Higher SBP in offspring of mothers with greater GWG, though not significant (β=0.2mmHg/0.1kg, 95% CI: -0.2-0.6); no significant association between maternal GWG and offspring DBP (β: -0.0mmHg/0.1kg, 95% CI: -0.3-0.2) or hypertension (OR: 1.0, 95% CI: 0.9-1.2); significant interaction of GWG and sex on adult blood pressure (all p<0.05).  *Adjusted for offspring age, sex, maternal age, education, parity, cigarette smoking and pre-pregnancy BMI. |
| --- | --- | --- | --- | --- |
| Hochner et al (2012)[30]  Israel  Cohort | N=1130, 32 years  Participants of the Jerusalem Perinatal Study (JPS) Family Follow-up study, singletons and term births without congenital malformations. | Maternal GWG  Maternal prepregnancy BMI (ppBMI) (kg/m2) | SBP (mmHg)  DBP (mmHg)  LDL cholesterol (mmol/L)  HDL cholesterol (mmol/L)  Triglycerides (TG) (mmol/L)  Plasma glucose (mmol/L/kg)  Insulin (pmol/L/kg) | Significant positive association between maternal GWG and offspring SBP (β = 0.206mmHg/kg, 95% CI: 0.003-0.408, p=0.047) and DBP (borderline) (β = 0.174, 95% CI: -0.004-0.353, p=0.055), and between mppBMI and offspring SBP (β = 0.441mmHg/kg/m2, 95%CI: 0.149-0.732, p=0.003) and DBP (β = 0.287, 95% CI = 0.05`-0.523, p=0.017), but all associations attenuated to null following further adjustment for offspring concurrent BMI; significant interaction of GWG with sex on blood pressure (SBP: p_interaction_ = 0.004, DBP: p_interaction_ = 0.001).  Significant positive association of GWG with TG (β = 0.005mmol/L/kg/m2, 95% CI: 0.0001-0.009, p=0.044), but not with LDL (p=0.429) or HDL (p=0.164); significant negative association of mppBMI with HDL (β = -0.010, 95% CI: -0.019- -0.0007, p=0.003) and TG (β = 0.007mmol/L/kg/m2, 95% CI: 0.001-0.012, p=0.02), but all significant associations attenuated to null following adjustment for concurrent offspring BMI.  No significant association of GWG with plasma glucose (β = 0.005mmol/L/kg, 95% CI: -0.008-0.019, p=0.48) or insulin (β = -0.003pmol/L/kg, 95% CI: -0.010-0.017, p=0.639); significant positive association of mppBMI with plasma insulin (β = 0.008pmol/L/kg/m2, 95% CI: 0.002-0.014, p=0.007), but not plasma glucose (β = -0.001mmol/L/kg/m2, 95% CI: -0.019-0.016, p=0.875), although all significant associations attenuated to null following further adjustment for offspring concurrent BMI.  *Adjusted for ethnicity, sex, mppBMI or maternal GWG, maternal parity, maternal age, maternal smoking, maternal SES, maternal medical condition, birth weight, gestational week, and offspring characteristics at 32 years (smoking status, physical activity, years of education). |
| Hrolfsdottir et al (2015)[31]  Denmark  Cohort | N=308, 39% male, 19-20 years  Offspring of normal weight mothers attending main midwife clinic in city of Aarhus, Denmark. | Maternal GWG during first 30 weeks gestation (GWG30) and total maternal GWG | SBP (mmHg)  DBP (mmHg)  Total cholesterol  LDL cholesterol (%)  HDL cholesterol (%)  Triglycerides (TG) (%)  Fasting plasma glucose  Fasting plasma insulin  HOMA-IR | Significant positive association between GWG30 and offspring SBP (β = 0.3mmHg/kg, 95% CI: 0.0, 0.6, p=0.03) for both sexes combined, but not when analysed separately by sex, although effect size greater for males (β = 0.4mmHg/kg, 95% CI: -0.1-0.9, p=0.09) than females (β= 0.2mmHg/kg, 95% CI: -0.2-0.5, p=0.3); significant positive association between GWG30 and DBP for males (β = 0.4mmHg/kg, 95% CI: -.0-0.8, p=0.03) but not females (β= -0.0mmHg/kg, 95% CI:-0.2-0.3, p=0.92) or both sexes combined (p=0.12); no significant association between total GWG and SBP or DBP (P>0.05).  Significant inverse association between GWG30 and total cholesterol and LDL cholesterol for both sexes combined (β = -0.9%/kg, p<0.01; β =-1.3%/kg, p<0.01) and for women (β = -1.3%/kg; β = -2.2%/kg, p<0.01) but not men (p=0.81; p=0.85) (interactions of GWG with sex significant, p<0.05); significant inverse association between GWG and HDL cholesterol in men (β = -1.1%, p=0.05) but not women (p=0.8); no significant association between GWG and TG overall, or in either sex.  Significant positive association between GWG30 and plasma insulin and HOMA-IR in men (β = 3.7%/kg, 95% CI: 1.4-6.2, p<0.01; β = 3.4%/kg, 95% CI: 0.8-6.0, p=0.01) but not women (p=0.82; p=0.94) or both sexes combined (p=0.09; p=0.14); no significant association between GWG30 and fasting glucose (p=0.95) for men and women separately; significant positive association between total GWG and plasma insulin for men (p for trend = 0.05) and both sexes combined (p for trend = 0.05) but not for women (p for trend = 0.23); significant positive association between total GWG and HOMA-IR for both sexes combined (p for trend = 0.05, but not for men or women separately (p>0.05).  *Adjusted for maternal pre-pregnancy BMI, age, parity, smoking status, educational level, offspring's sex, whether offspring think their father is overweight |
| Scheers-Andersson et al (2015)[32]  Sweden  Cohort | N=9816, 100% male, mean age 18.3 years  9186 full brothers (4908 brother pairs) who underwent military conscription induction tests from 2000 to 2008. | Maternal GWG (weight at delivery – weight ≈10 weeks gestation). | SBP (mmHg)  DBP (mmHg)  Hypertension (%) | No significant association between GWG and offspring blood pressure or risk of hypertension within siblings nor between unrelated family members (p>0.05).  *Adjusted for maternal age at birth, birth year, gestational age, early-pregnancy BMI, maternal education and parity, offspring’s age at conscription and conscription centre. |
| Mi et al (2000)  China  Cross-sectional | N=627, 49% male, aged 41-47 years  Men and women born in Beijing, China approximately 45 years ago, whose mothers’ heights and weights during pregnancy were recorded by the Peking Union Medical College Hospital. | Maternal BMI at 15 weeks and 38 weeks of pregnancy  Maternal gestational weight gain | SBP (mmHg)  DBP (mmHg)  Total cholesterol (mmol/L)  LDL cholesterol (mmol/L)  HDL cholesterol (mmol/L)  Triglyceride (TG) level (mmol/L)  Fasting plasma glucose (mmol/L)  120min glucose (mmol/L)  Fasting insulin (mmol/L)  120min insulin (mmol/L) | No significant association between maternal BMI (at either 15 weeks or 38 weeks gestation) and offspring SBP or DBP. No significant association between maternal GWG and offspring blood pressure.  Statistically significant inverse association between maternal BMI at 15 weeks gestation and total cholesterol (5.21 for BMI≤19.2 vs. 4.77 for BMI>22.3, p=0.02) and LDL cholesterol (3.14 for BMI≤19.2 vs. 2.76 for BMI>22.3, p=0.01), but not HDL cholesterol (p>0.02) or TG level (p=0.06); no significant association between maternal BMI at 38 weeks gestation and total cholesterol (p=0.1), LDL cholesterol (p=0.2), HDL cholesterol (>0.2) or TG (p>0.2). No significant association between maternal GWG and any blood lipid analysed.  Significant inverse association between maternal BMI and offspring 120min glucose level at 15 weeks gestation (7.4 for BMI≤19.2 vs. 5.6 for BMI>22.3, p=0.008) and 38 weeks gestation (7.6 for BMI≤19.2 vs. 5.7 for BMI>22.3, p=0.003), and offspring 120min insulin level at 15 weeks gestation (399 for BMI≤19.2 vs. 181 for BMI>22.3, p=0.02) and 38 weeks gestation (304 for BMI≤19.2 vs. 177 for BMI>22.3, p=0.007), but association between maternal BMI and offspring fasting glucose (p>0.2, p=0.2) and fasting insulin levels (p=0.07, p=0.1) not significant. No significant association between maternal GWG and offspring blood glucose or insulin levels.  *Adjusted for sex and BMI. |
| Loos et al (2002)  Belgium  Cross-sectional | N=800, 49% male, 18-34 years  Sample of twin pairs from population-based East Flanders Prospective Twin Survey. | Maternal pre-pregnancy BMI  Maternal BMI increase during pregnancy (BMI at end of pregnancy minus BMI before pregnancy) | Fasting plasma glucose (mmol/L)  Fasting plasma proinsulin (pmol/L)  Fasting plasma insulin (mmol/L)  Homeostatic model assessment-insulin resistance (HOMA-IR)  β-Cell function (HOMA) | Significant inverse association between maternal pre-pregnancy BMI and female offspring fasting insulin (β=-2.7% per 1kg/m^2^ , p=0.003) and insulin resistance (β=-2.5% per 1kg/m^2^ , p=0.005), but not for male offspring (β=0.1% per 1kg/m^2^ , p=0.82; (β=0.0% per 1kg/m^2^ , p=0.99); significant inverse association for both sexes between maternal pre-pregnancy BMI (β=-3.1% per 1kg/m^2^ , p=0.003) and proinsulin and β-cell function (β=-2.1% per 1kg/m^2^ , p=0.005), but no significant association with fasting plasma glucose (β=0.2% per 1kg/m^2^ , p=0.17); no significant association between maternal weight gain and offspring insulin resistance (no mention of other outcomes).  *Adjusted for age, sex, gestational age, adult BMI. |
|  |  |  |  |  |
| Webb et al (2005)[28]  Guatemala  Longitudinal | N=450, 50% male, 21-29 years  Rural-born Guatemalan adults who were part of the INCAP longitudinal study, which involved in a randomized trial of nutritional supplementation of their mothers during pregnancy and during their early childhoods from 1969-1977. | Supplement type consumed by mother (Atole: provided protein, micronutrients and 3.80MJ/L or Fresco: provided only micronutrients and 1.35MJ/L.  Prenatal supplement intake (MJ/day)  Maternal GWG  Maternal non-pregnant BMI (≥5.5 months post-partum)  Maternal height | SBP (mmHg)  DBP (mmHg) | No association between protein-energy supplementation and adult offspring SBP or DBP; weak though not significant inverse associations between GWG and offspring blood pressure (Atole SBP: β = -0.89mmHg/kg/month, 95% CI: -2.97-1.11, p=0.38, Fresco SBP: β = 0.35mmHg/kg/month, 95% CI: -1.98-2.58, p=0.79; Atole DBP: β = -0.33mmHg/kg/month, 95% CI: -1.97-1.31, p=0.69, Fresco DBP: β = -0.48mmHg/kg/month, 95% CI: -2.15-1.20, p=0.57); no significant association between maternal non-pregnant BMI and offspring blood pressure in either supplement group (Atole SBP: p=-0.97, Fresco SBP: p=0.09, Atole DBP: p=0.85, Fresco DBP: p=0.15); significant positive association between maternal height and offspring SBP in Atole group only (β = 0.22mmHg/cm, 95% CI: -0.002-0.45, p=0.05), no significant association between maternal height and DBP in either supplement group.  *Adjusted for sex, SES, village size and supplement group at birth, attained education, adult age, physical activity and migration status, BMI and WHR, alcohol consumption and smoking status. |

**Maternal weight/BMI**

| Hochner et al (2012)[30]  Israel  Cohort | N=1130, 32 years  Participants of the Jerusalem Perinatal Study (JPS) Family Follow-up study, singletons and term births without congenital malformations. | Maternal GWG  Maternal prepregnancy BMI (ppBMI) (kg/m2) | SBP (mmHg)  DBP (mmHg)  LDL cholesterol (mmol/L)  HDL cholesterol (mmol/L)  Triglycerides (TG) (mmol/L)  Plasma glucose (mmol/L/kg)  Insulin (pmol/L/kg) | Significant positive association between maternal GWG and offspring SBP (β = 0.206mmHg/kg, 95% CI: 0.003-0.408, p=0.047) and DBP (borderline) (β = 0.174, 95% CI: -0.004-0.353, p=0.055), and between mppBMI and offspring SBP (β = 0.441mmHg/kg/m2, 95%CI: 0.149-0.732, p=0.003) and DBP (β = 0.287, 95% CI = 0.05`-0.523, p=0.017), but all associations attenuated to null following further adjustment for offspring concurrent BMI; significant interaction of GWG with sex on blood pressure (SBP: p_interaction_ = 0.004, DBP: p_interaction_ = 0.001).  Significant positive association of GWG with TG (β = 0.005mmol/L/kg/m2, 95% CI: 0.0001-0.009, p=0.044), but not with LDL (p=0.429) or HDL (p=0.164); significant negative association of mppBMI with HDL (β = -0.010, 95% CI: -0.019- -0.0007, p=0.003) and TG (β = 0.007mmol/L/kg/m2, 95% CI: 0.001-0.012, p=0.02), but all significant associations attenuated to null following adjustment for concurrent offspring BMI.  No significant association of GWG with plasma glucose (β = 0.005mmol/L/kg, 95% CI: -0.008-0.019, p=0.48) or insulin (β = -0.003pmol/L/kg, 95% CI: -0.010-0.017, p=0.639); significant positive association of mppBMI with plasma insulin (β = 0.008pmol/L/kg/m2, 95% CI: 0.002-0.014, p=0.007), but not plasma glucose (β = -0.001mmol/L/kg/m2, 95% CI: -0.019-0.016, p=0.875), although all significant associations attenuated to null following further adjustment for offspring concurrent BMI.  *Adjusted for ethnicity, sex, mppBMI or maternal GWG, maternal parity, maternal age, maternal smoking, maternal SES, maternal medical condition, birth weight, gestational week, and offspring characteristics at 32 years (smoking status, physical activity, years of education). |
| --- | --- | --- | --- | --- |
| Mi et al (2000)  China  Cross-sectional | N=627, 49% male, aged 41-47 years  Men and women born in Beijing, China approximately 45 years ago, whose mothers’ heights and weights during pregnancy were recorded by the Peking Union Medical College Hospital. | Maternal BMI at 15 weeks and 38 weeks of pregnancy  Maternal gestational weight gain | SBP (mmHg)  DBP (mmHg)  Total cholesterol (mmol/L)  LDL cholesterol (mmol/L)  HDL cholesterol (mmol/L)  Triglyceride (TG) level (mmol/L)  Fasting plasma glucose (mmol/L)  120min glucose (mmol/L)  Fasting insulin (mmol/L)  120min insulin (mmol/L) | No significant association between maternal BMI (at either 15 weeks or 38 weeks gestation) and offspring SBP or DBP. No significant association between maternal GWG and offspring blood pressure.  Statistically significant inverse association between maternal BMI at 15 weeks gestation and total cholesterol (5.21 for BMI≤19.2 vs. 4.77 for BMI>22.3, p=0.02) and LDL cholesterol (3.14 for BMI≤19.2 vs. 2.76 for BMI>22.3, p=0.01), but not HDL cholesterol (p>0.02) or TG level (p=0.06); no significant association between maternal BMI at 38 weeks gestation and total cholesterol (p=0.1), LDL cholesterol (p=0.2), HDL cholesterol (>0.2) or TG (p>0.2). No significant association between maternal GWG and any blood lipid analysed.  Significant inverse association between maternal BMI and offspring 120min glucose level at 15 weeks gestation (7.4 for BMI≤19.2 vs. 5.6 for BMI>22.3, p=0.008) and 38 weeks gestation (7.6 for BMI≤19.2 vs. 5.7 for BMI>22.3, p=0.003), and offspring 120min insulin level at 15 weeks gestation (399 for BMI≤19.2 vs. 181 for BMI>22.3, p=0.02) and 38 weeks gestation (304 for BMI≤19.2 vs. 177 for BMI>22.3, p=0.007), but association between maternal BMI and offspring fasting glucose (p>0.2, p=0.2) and fasting insulin levels (p=0.07, p=0.1) not significant. No significant association between maternal GWG and offspring blood glucose or insulin levels.  *Adjusted for sex and BMI. |
| Loos et al (2002)  Belgium  Cross-sectional | N=800, 49% male, 18-34 years  Sample of twin pairs from population-based East Flanders Prospective Twin Survey. | Maternal pre-pregnancy BMI  Maternal BMI increase during pregnancy (BMI at end of pregnancy minus BMI before pregnancy) | Fasting plasma glucose (mmol/L)  Fasting plasma proinsulin (pmol/L)  Fasting plasma insulin (mmol/L)  Homeostatic model assessment-insulin resistance (HOMA-IR)  β-Cell function (HOMA) | Significant inverse association between maternal pre-pregnancy BMI and female offspring fasting insulin (β=-2.7% per 1kg/m^2^ , p=0.003) and insulin resistance (β=-2.5% per 1kg/m^2^ , p=0.005), but not for male offspring (β=0.1% per 1kg/m^2^ , p=0.82; (β=0.0% per 1kg/m^2^ , p=0.99); significant inverse association for both sexes between maternal pre-pregnancy BMI (β=-3.1% per 1kg/m^2^ , p=0.003) and proinsulin and β-cell function (β=-2.1% per 1kg/m^2^ , p=0.005), but no significant association with fasting plasma glucose (β=0.2% per 1kg/m^2^ , p=0.17); no significant association between maternal weight gain and offspring insulin resistance (no mention of other outcomes).  *Adjusted for age, sex, gestational age, adult BMI. |
| Webb et al (2005)[28]  Guatemala  Longitudinal | N=450, 50% male, 21-29 years  Rural-born Guatemalan adults who were part of the INCAP longitudinal study, which involved in a randomized trial of nutritional supplementation of their mothers during pregnancy and during their early childhoods from 1969-1977. | Supplement type consumed by mother (Atole: provided protein, micronutrients and 3.80MJ/L or Fresco: provided only micronutrients and 1.35MJ/L.  Prenatal supplement intake (MJ/day)  Maternal GWG  Maternal non-pregnant BMI (≥5.5 months post-partum)  Maternal height | SBP (mmHg)  DBP (mmHg) | No association between protein-energy supplementation and adult offspring SBP or DBP; weak though not significant inverse associations between GWG and offspring blood pressure (Atole SBP: β = -0.89mmHg/kg/month, 95% CI: -2.97-1.11, p=0.38, Fresco SBP: β = 0.35mmHg/kg/month, 95% CI: -1.98-2.58, p=0.79; Atole DBP: β = -0.33mmHg/kg/month, 95% CI: -1.97-1.31, p=0.69, Fresco DBP: β = -0.48mmHg/kg/month, 95% CI: -2.15-1.20, p=0.57); no significant association between maternal non-pregnant BMI and offspring blood pressure in either supplement group (Atole SBP: p=-0.97, Fresco SBP: p=0.09, Atole DBP: p=0.85, Fresco DBP: p=0.15); significant positive association between maternal height and offspring SBP in Atole group only (β = 0.22mmHg/cm, 95% CI: -0.002-0.45, p=0.05), no significant association between maternal height and DBP in either supplement group.  *Adjusted for sex, SES, village size and supplement group at birth, attained education, adult age, physical activity and migration status, BMI and WHR, alcohol consumption and smoking status. |

**Maternal dietary intake**

| Campbell et al (1996)[33]  UK  Cohort | N=253, mean age 40.6 years  Individuals born in Aberdeen Maternity Hospital between 1948-1954, whose mothers had taken part in a survey of diet in late pregnancy. | Maternal intake of protein, animal protein, fat, carbohydrate, calcium, vitamin A, thiamine, riboflavin, niacin, vitamin C in 7 month of pregnancy | SBP (mmHg)  DBP (mmHg) | No significant associations between intake of any individual nutrient and offspring blood pressure, but for animal protein intakes ≤50g, increasing carbohydrate intake associated with significantly higher offspring SBP (β = +3.0mmHg/100g increase carbohydrate, p=0.02) and DBP (borderline) (β = +1.9/100g increase carbohydrate, p=0.06), while for animal protein intakes >50g, increasing carbohydrate intake associated with significantly lower SBP (β = -11.2mmHg/100g increase carbohydrate, p=0.004) and DBP (β = -7.7mmHg/100g increase carbohydrate, p=0.001).  *Adjusted for birthweight and mothers’ blood pressure. |
| --- | --- | --- | --- | --- |
| Roseboom et al (2001) (cite)  Netherlands  Cohort | N=739, 48% male, ˜50 years  739 individuals who were born at term as singletons in Wilhelmina Gasthuis University Hospital in Amsterdam between 1st November 1943 and 28th February 1947. | Maternal protein/carbohydrate ratio in 3rd trimester | SBP (mmHg)  DBP (mmHg) | No significant association between total caloric, protein carbohydrate or fat intake during any week of gestation and offspring SBP or DBP; significant negative association between protein/carbohydrate intake in 3rd trimester and adult offspring SBP (126.1mmHg for ≤15% protein/carbohydrate vs. 124.3mmHg for >20% protein/carbohydrate, p for trend=0.02) in both exposed and non-exposed offspring; no significant association for DBP.  *Adjusted for sex |
| Shiell et al (2001) [37]  UK  Cohort | N=626, 44% male, 27-30 years  Individuals born in Motherwell, Scotland, whose mothers' had taken part in a dietary intervention in which they were advised to each 0.45kg of red meat per day and to avoid carbohydrate-rich foods during pregnancy. | Maternal consumption of meat and fish (plus low carbohydrate) during pregnancy post 20 weeks gestation | SBP (mmHg)  DBP (mmHg) | Significant positive association between maternal meat and fish consumption and offspring SBP (β = 0.19mmHg/portions/week, 95% CI: 0.004-0.35, p=0.02) and between maternal fish (but not meat) consumption and offspring DBP (β = 1.00mmHg/portions/week, 95% CI: 0.18-1.82, p=0.02), with greatest effect of meat and fish consumption on SBP in offspring of mothers with low green vegetable intake (<7 green vegetable portions per week: β = 0.26mmHg/meat and fish portions/week, 95% CI: 0.03-0.50, p=0.03; ≥7 portions green vegetables per week: β = 0.16mmHg/meat and fish portions/week, 95% CI: -0.06-0.38, p=0.16).  * Adjusted for offspring sex, BMI, alcohol consumption, cuff size. |
| Conlisk et al (2004)  Guatemala  Longitudinal | N=429, 49% male, mean age 24.4 years  Rural-born Guatemalan adults who were part of the INCAP longitudinal study, which involved in a randomized trial of nutritional supplementation of their mothers during pregnancy and during their early childhoods from 1969-1977. | Supplement type consumed by mother mother (Atole: provided protein, micronutrients and 3.80MJ/L or Fresco: provided only micronutrients and 1.35MJ/L.  Prenatal energy supplementation (MJ/L)  Volume of prenatal supplementation (mmol/L) | Fasting plasma glucose level (mmol/L) | No significant association between supplement type or prenatal energy supplementation and fasting plasma glucose in men; in women exposure to Atole associated with significantly lower fasting plasma glucose compared with Fresco (difference=0.29mmol/L, p=0.03), although not significant after adjusting for adult BMI; there was a significant inverse association between prenatal energy intake from supplement and adult fasting plasma glucose (β=-0.39±0.16mmol/L/MJ/d (p=0.02) and between volume of prenatal supplementation (β=-0.87±0.42mmol/L/L/d (p=0.04) and adult fasting plasma glucose in women; no significant interactions between supplement type and either prenatal energy supplementation or volume of prenatal supplementation affecting fasting plasma glucose in either men or women.  *Adjusted for gestational age, socioeconomic status, village size, adult age, energy intake, physical activity (women only), and migration status (women only). Analyses of exposures other than supplement type were also controlled for supplement type. |
| Webb et al (2005)[28]  Guatemala  Longitudinal | N=450, 50% male, 21-29 years  Rural-born Guatemalan adults who were part of the INCAP longitudinal study, which involved in a randomized trial of nutritional supplementation of their mothers during pregnancy and during their early childhoods from 1969-1977. | Supplement type consumed by mother (Atole: provided protein, micronutrients and 3.80MJ/L or Fresco: provided only micronutrients and 1.35MJ/L.  Prenatal supplement intake (MJ/day)  Maternal GWG  Maternal non-pregnant BMI (≥5.5 months post-partum)  Maternal height | SBP (mmHg)  DBP (mmHg) | No association between protein-energy supplementation and adult offspring SBP or DBP; weak though not significant inverse associations between GWG and offspring blood pressure (Atole SBP: β = -0.89mmHg/kg/month, 95% CI: -2.97-1.11, p=0.38, Fresco SBP: β = 0.35mmHg/kg/month, 95% CI: -1.98-2.58, p=0.79; Atole DBP: β = -0.33mmHg/kg/month, 95% CI: -1.97-1.31, p=0.69, Fresco DBP: β = -0.48mmHg/kg/month, 95% CI: -2.15-1.20, p=0.57); no significant association between maternal non-pregnant BMI and offspring blood pressure in either supplement group (Atole SBP: p=-0.97, Fresco SBP: p=0.09, Atole DBP: p=0.85, Fresco DBP: p=0.15); significant positive association between maternal height and offspring SBP in Atole group only (β = 0.22mmHg/cm, 95% CI: -0.002-0.45, p=0.05), no significant association between maternal height and DBP in either supplement group.  *Adjusted for sex, SES, village size and supplement group at birth, attained education, adult age, physical activity and migration status, BMI and WHR, alcohol consumption and smoking status. |
| Danielsen et al (2013)  Denmark  Cohort | N=428, 47% male, age 20 years  Sample of subjects from the Danish Fetal Origin Cohort 1988. | Maternal glycemic index (GI) in gestational week 30 (2^nd^ trimester)  Maternal glycemic load (GL) in gestational week 30 (2^nd^ trimester) | SBP (mmHg)  DBP (mmHg)  Total cholesterol (mmol/L)  LDL cholesterol (mmol/L)  HDL cholesterol (mmol/L)  Triglycerides (mmol/L)  Fasting plasma glucose (mmol/L)  Fasting plasma insulin (mmol/L)  Homeostatic model assessment-insulin resistance (HOMA-IR) | No significant association between maternal GI and SBP or DBP in both sexes or males and females separately (p>0.05); significant negative association between maternal GL and SBP in females (β=-0.46/10U GI increase, 95% CI: -0.91 - -0.01, p<0.05) but not males (p>0.05).  Significant positive association between maternal GI and total cholesterol in both sexes (β=1.03/10U GI increment, 95% CI: 1.00, 1.06, p=0.05) and in females (β=1.05/10U GI increment, 95% CI: 1.01, 1.09, p=0.01) but not in males (p>0.05); borderline significant association between maternal GI and HDL cholesterol in both sexes (β=1.03/10U GI increment, 95% CI: 1.00, 1.06, p=0.09) and in females (β=1.04/10U GI increment, 95% CI: 1.01, 1.09, p=0.06) but not in males (p>0.05); no significant associations observed between maternal GI and LDL cholesterol or for GL and any blood lipid variable.  No significant association between maternal GI and fasting plasma glucose in both sexes ( p=0.18), or males (p>0.05) and females (p=0.76) separately; significant positive association between maternal GI and fasting plasma in both sexes insulin (β=1.09, 95% CI: 1.01-1.16,p=0.02) and borderline significant in females (β=1.08, 95% CI: 0.99-1.19, p=0.07) but no significant association in males; significant positive association between maternal GI and HOMA-IR in both sexes (β=1.09, 95% CI: 1.01-1.16,p=0.02) but not males and females separately; no significant associations observed for GL.  *Adjusted for maternal smoking during pregnancy, height, pre-pregnancy BMI, education, energy intake, and offspring’s sex and ambient level of physical activity. |
| Rytter et al (2011)[40]  Denmark  RCT | N=243, 18-19 years  243 offspring of mothers visiting main midwife clinic in city of Aarhus, Denmark. | Fish oil supplementation (marine PUFA) from gestational week 30 | Total cholesterol  LDL cholesterol (mmol/L)  HDL cholesterol (mmol/L)  Triacylglycerols (TAG) (mmol/L)  Apolipoprotein A-I (Apo-AI) (g/L)  Apolipoprotein B (Apo-B) (g/L) | No significant association between fish oil supplementation and total, LDL, or HDL cholesterol, TAG, Apo-A1 or Apo-B in offspring (p>0.05)  *Adjusted for sex. |
| Rytter et al (2012)[51]  Denmark  RCT | N=180, 19 years  180 offspring of mothers visiting main midwife clinic in city of Aarhus, Denmark, follow-up of RCT from 1990. | Fish oil supplementation (marine n-3 PUFA) during pregnancy from gestational week 30 to delivery | SBP (mmHg)  DBP (mmHg) | No significant association between maternal fish oil supplementation and offspring SBP (difference for fish oil vs. olive oil reference group = 2.0mmHg (95% CI: -1, 4) or DBP (difference = 1.0mmHg, 95% CI: 0-3)  *Adjusted for offspring sex, smoking and maternal SBP (or DBP) in gestational week 30 |
| Rytter et al (2013)  Denmark  Cohort | N=443, 39% male, 19-20 years  Offspring of women making visits for routine antenatal care to a specific midwife centre in the city of Aarhus, Denmark from April 1988 to January 1989. | Maternal intake of energy-adjusted marine n-3 PUFA during 2nd trimester of pregnancy | SBP (mmHg)  DBP (mmHg)  Total cholesterol  LDL cholesterol (mmol/L)  HDL cholesterol (mmol/L)  Triacylglycerols (TAG) (mmol/L)  Apolipoprotein A-I (Apo-AI) (g/L)  Apolipoprotein B (Apo-B) (g/L)  Plasma glucose (mmol/L)  Plasma insulin (pmol/L)  Homeostatic model assessment-insulin resistance (HOMA-IR) | No significant association between maternal intake of n-3 PUFA during pregnancy and offspring SBP (0.0% difference between Q5 vs. Q1, p for trend=0.854) or DBP (0.0% difference Q5 vs. Q1, p for trend=0.484).  No significant association between maternal intake of energy-adjusted n-3 PUFA and offspring total, LDL or HDL cholesterol, or Apo-A1, Apo-B, or TAG concentrations (P>0.05).  No significant association between maternal intake of n-3 PUFA during pregnancy and offspring plasma insulin (p for trend=0.144), glucose (p=0.998), or HOMA-IR (p=0.164).  *Adjusted for maternal pre-pregnancy BMI, maternal education, smoking during pregnancy, maternal age, parity, energy intake and sex |
| Macleod et al (2013)  UK  RCT | N=118  Offspring of South Asian mothers who participated in two trials of nutritional supplementation during pregnancy (protein/energy/vitamins, energy/vitamins or vitamins only) at Sorrento Maternity Hospital in Birmingham, UK either unselected or selected based on under-nutritional status. | Maternal protein and carbohydrate supplementation (vitamins only = control group) | SBP (mmHg)  DBP (mmHg)  Total cholesterol (mmol/L)  HDL cholesterol (mmol/L)  Triglycerides (mmol/L)  Fasting plasma glucose (mmol/L)  120 mins post load glucose (mmol/L0  Fasting plasma insulin (pmol/L) | No consistent significant pattern of association of nutritional supplementation (protein/carbohydrate plus vitamins relative to vitamins only) and blood pressure in offspring of either unselected mothers at 18 weeks or underweight selected mothers at 28 weeks gestation.  No consistent significant pattern of association of nutritional supplementation (protein/carbohydrate plus vitamins relative to vitamins only) and blood lipids in offspring of either unselected mothers at 18 weeks gestation or underweight selected mothers at 28 weeks gestation.  No consistent significant pattern of association of nutritional supplementation (protein/carbohydrate plus vitamins relative to vitamins only) and blood glucose level/metabolism in offspring of either unselected mothers at 18 weeks gestation or underweight selected mothers at 28 weeks gestation  *Adjusted for age, sex and adult adiposity. |
